# Structural Characterization of Fluorescent Proteins Using Tunable Femtosecond Stimulated Raman Spectroscopy

**DOI:** 10.3390/ijms241511991

**Published:** 2023-07-26

**Authors:** Cheng Chen, J. Nathan Henderson, Dmitry A. Ruchkin, Jacob M. Kirsh, Mikhail S. Baranov, Alexey M. Bogdanov, Jeremy H. Mills, Steven G. Boxer, Chong Fang

**Affiliations:** 1 Department of Chemistry, Oregon State University, 153 Gilbert Hall, Corvallis, OR 97331, USA; chenc9@oregonstate.edu; 2 Center for Molecular Design and Biomimetics, The Biodesign Institute, Arizona State University, Tempe, AZ 85287, USA; nhenderson@asu.edu (J.N.H.); jeremy.mills@asu.edu (J.H.M.); 3 Shemyakin-Ovchinnikov Institute of Bioorganic Chemistry, Russian Academy of Sciences, Ulitsa Miklukho-Maklaya, 16/10, 117997 Moscow, Russia; evro702@icloud.com (D.A.R.); baranovmikes@ibch.ru (M.S.B.); noobissat@yandex.ru (A.M.B.); 4 Department of Chemistry, Stanford University, Stanford, CA 94305, USA; jkirsh@stanford.edu (J.M.K.); sboxer@stanford.edu (S.G.B.); 5 Laboratory of Medicinal Substances Chemistry, Institute of Translational Medicine, Pirogov Russian National Research Medical University, Ostrovitianov 1, 117997 Moscow, Russia; 6 School of Molecular Sciences, Arizona State University, Tempe, AZ 85287, USA

**Keywords:** green fluorescent protein, red fluorescent protein, femtosecond stimulated Raman spectroscopy, resonance structures, chromophore–environment interactions, *cis* and *trans* conformations

## Abstract

The versatile functions of fluorescent proteins (FPs) as fluorescence biomarkers depend on their intrinsic chromophores interacting with the protein environment. Besides X-ray crystallography, vibrational spectroscopy represents a highly valuable tool for characterizing the chromophore structure and revealing the roles of chromophore–environment interactions. In this work, we aim to benchmark the ground-state vibrational signatures of a series of FPs with emission colors spanning from green, yellow, orange, to red, as well as the solvated model chromophores for some of these FPs, using wavelength-tunable femtosecond stimulated Raman spectroscopy (FSRS) in conjunction with quantum calculations. We systematically analyzed and discussed four factors underlying the vibrational properties of FP chromophores: sidechain structure, conjugation structure, chromophore conformation, and the protein environment. A prominent bond-stretching mode characteristic of the quinoidal resonance structure is found to be conserved in most FPs and model chromophores investigated, which can be used as a vibrational marker to interpret chromophore–environment interactions and structural effects on the electronic properties of the chromophore. The fundamental insights gained for these light-sensing units (e.g., protein active sites) substantiate the unique and powerful capability of wavelength-tunable FSRS in delineating FP chromophore properties with high sensitivity and resolution in solution and protein matrices. The comprehensive characterization for various FPs across a colorful palette could also serve as a solid foundation for future spectroscopic studies and the rational engineering of FPs with diverse and improved functions.

## 1. Introduction

**Figure 1:**
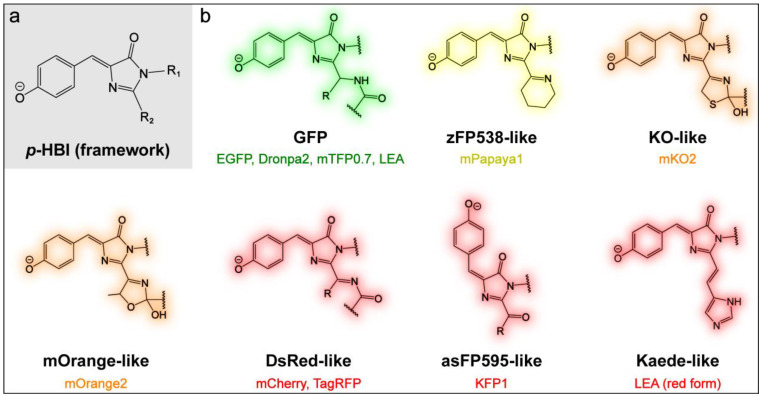
Illustrations of the chemical structures of the (**a**) model chromophore of FPs, *p*-HBI, and (**b**) chromophore moieties of various FPs. The names in black indicate the FPs that were first found for their respective category by the pertinent chromophore structure with characteristic emission hues. Note that the coloring highlights the chromophore structure without an exact correlation to the conjugation size (see main text for details about the conjugation extension at the I-ring R_2_ site). The FPs studied in this work are color-coded and labeled under each representative chromophore structure.

Fluorescent proteins (FPs) have remarkably advanced molecular and cellular biology over the past few decades. The early serendipitous and later more informed discovery of various FPs from nature that emit different colors of fluorescence has opened the avenue for wide varieties of imaging applications through protein engineering and also prompted extensive mechanistic investigations [1,2,3,4,5]. The photophysical and photochemical properties of FPs such as color and fluorescence quantum yield (FQY) originate from their autocatalytically formed chromophores that are located at the center of the β-barrel structure, and greatly depend on the interactions between the chromophore and its surrounding protein environment. The wild-type green fluorescent protein (GFP) found in the jellyfish *Aequorea victoria* and its green derivatives/relatives share the same chromophore structure of *p*-hydroxybenzylidene-imidazolinone (*p*-HBI, Figure 1a; note the R_1_ and R_2_ substituents therein), formed from the Ser65-Tyr66-Gly67 tripeptide (commonly referred to as the SYG chromophore), and its green fluorescence arises from the deprotonated anionic form of the chromophore [2,6,7]. The emission wavelength is sensitive to the local environment due to the photoinduced intramolecular charge transfer of the chromophore, but it is still limited to the cyan-to-green wavelength region without further extension of the chromophore conjugation. Subsequently, the color palette of emission has been expanded from green to yellow, orange, and red with the discovery (both from Hydrozoan and Anthozoan species) and engineering of various FPs possessing conjugation-extended chromophores (i.e., a larger quantum “box” with closer energy spacing) [5]. The red fluorescent protein (RFP) has drawn particular attention due to the advantages of long spectral wavelengths in imaging applications such as deep tissue penetration, reduced autofluorescence, and decreased phototoxicity. The red chromophores in most RFPs are formed through autocatalytic mechanisms, although the detailed reaction steps remain debated. They can also be generated via photoconversion, and this class of RFPs (e.g., Kaede and EosFP) has the same chromophore derived from the His-Tyr-Gly tripeptide (i.e., the HYG chromophore) [8,9,10].

Deciphering the working mechanisms of FPs requires the structural characterization of the chromophore in its local environment. A commonly used method is X-ray crystallography which can reveal the structure of an FP chromophore as well as its interactions, such as hydrogen (H)-bonding with nearby residues, water, and ions. The information extracted from the crystal structure is straightforward and can provide important insights into the photophysical and photochemical properties of the chromophore. However, several limitations and concerns have been raised about this method. Besides the intrinsic requirement for protein crystals or microcrystals, X-ray diffraction is insensitive to hydrogen atoms at the typical resolution available, and consequently structural information such as the chromophore protonation state is difficult to assess by this method alone [11,12,13]. It has also been debated about whether the FP structure in the crystalline state is the same as that in aqueous solution where FPs are usually studied or used for applications [14,15]. In fact, the way of preparing the crystal sometimes can alter the photophysical and photochemical properties of an FP. In a recent work on the *cis*-to-*trans* photoisomerization pathways of photoswitchable FPs, Chang et al. investigated a reversibly switchable enhanced GFP (rsEGFP2) variant containing a monochlorinated Ala-Tyr-Gly (AYG) chromophore, where the resultant structural symmetry breaking enables the differentiation between one-bond-flip and hula-twist mechanisms via X-ray crystallography [16,17]. The authors found that the exact pathway depends on the packing of the protein, which is experimentally controllable, that is, the more volume-demanding one-bond-flip pathway is favored in an expanded crystal lattice, while the hula-twist pathway prevails in a tighter packing configuration. The fact that the photoisomerization pathway is not intrinsic to the FP but varies with crystal packing conditions is interesting but also suggests that the crystal structures should be interpreted with caution.

Meanwhile, spectroscopic methods have often been used to characterize the properties of FPs. Electronic techniques such as steady-state UV/Visible and fluorescence spectroscopy can provide information about the absorption and emission wavelengths, protonation state, p*K*_a_, and FQY, etc., mainly due to the protein chromophore (i.e., the light-sensing unit or active site in these FPs) [2,18,19]. The details of the photoinduced processes in FPs such as excited-state proton transfer (ESPT) and *cis–trans* photoisomerization can be delineated by ultrafast electronic spectroscopic techniques with sufficient temporal resolution [6,20,21,22,23]. Compared with electronic spectroscopy, vibrational spectroscopy is intrinsically more sensitive to the chromophore structural changes and can thus offer structural information with deeper mechanistic insights [7,15,24,25,26,27]. For instance, the vibrational modes of GFP and its model chromophore are highly sensitive to the protonation state at the phenolic hydroxyl group. The ground-state infrared (IR) and Raman spectra have confirmed that the stretching frequencies of the methine bridge C=C and imidazolinone C=O bonds undergo redshifts upon deprotonation of the protonated (neutral) chromophore, reflective of the electron density redistribution that weakens the double-bond character [28,29,30]. For a given protonation state, particularly for the deprotonated (anionic) chromophore, GFP exhibits substantial frequency shifts from its model chromophore in solution for many vibrational modes, highlighting the role of the protein environment in modifying the structure and/or electron density distribution of the chromophore [26,28,31].

Compared to the rapid development of FPs with advanced functionalities via protein engineering methods, the mechanistic studies using vibrational spectroscopy are still lacking. As an example, photoswitchable fluorescent proteins (PsFPs) have gained momentum as useful tools for super-resolution microscopy [32,33,34]. However, detailed investigations of their working mechanisms via techniques like time-resolved serial femtosecond crystallography and IR/transient absorption spectroscopy have been limited to a few cases such as rsEGFP2 and Dronpa2 [25,35,36] despite the vast number of such FPs [37,38]. The complete photoswitching processes for most PsFPs remain underexplored, though the protein chromophore *cis–trans* photoisomerization and protonation state change have been considered to be the major reaction steps in common. The mechanistic insights of photoinduced processes are important in facilitating the bottom-up rational design of FPs with targeted and improved properties. The ultrafast vibrational spectroscopic techniques have started to demonstrate the capability of dissecting the structural dynamics of FPs in recent years [15,25,26,39,40]. Notably, RFPs with red-shifted emission wavelengths have been spectroscopically less investigated than GFPs but are increasingly drawing attention across bioimaging and biophysics communities. In view of the aforementioned advantages of RFP over GFP in practical applications, an increase in the mechanistic investigations of RFPs using vibrational spectroscopy is desirable.

In this work, we aim to benchmark the ground-state vibrational spectra for key series of FPs with different chromophore structures in their protein environments as well as for the synthesizable model chromophores in solution. The FPs investigated include GFPs (EGFP, Dronpa2, mTFP0.7, and LEA) [10,41,42,43], a yellow FP (mPapaya1) [44], orange FPs (mKO2 and mOrange2) [45,46,47,48], and RFPs (KFP1, mCherry, TagRFP, and the photoconverted LEA) [46,49,50,51], and their chromophore structures are illustrated in Figure 1b. We hereby focus on the deprotonated form of the FP chromophore, which is the primary emissive state, and implement wavelength-tunable femtosecond stimulated Raman spectroscopy (FSRS) for the acquisition of ground-state spectra without an actinic pump pulse [26,52]. Therefore, the incident laser beams involve a picosecond (ps) Raman pump and femtosecond (fs) Raman probe pair to stimulate the Raman scattering photons for signal detection in the frequency domain [31,53]. FSRS is highly suitable for the measurement of fluorescent systems like FPs because it is not only free from the fluorescence background that is a major issue for spontaneous Raman under pre- to on-resonance conditions, but it is also capable of taking advantage of the pertinent electronic absorption band (without any actinic pump) or stimulated emission band (when an actinic pump/photoexcitation pulse is used) to further improve the Raman signal-to-noise ratio. Our judicious choice of preresonance Raman pump (R_pu_) wavelengths allows the selective enhancement of the chromophore modes such that the rest of the protein residues (i.e., non-chromophore units) would not interfere due to their negligible Raman intensities. Through systematic comparisons of the vibrational marker bands via ground-state FSRS measurements, we discuss the roles of the (1) sidechain structure of the GFP model chromophore, (2) FP environment, (3) FP chromophore structure, and (4) FP chromophore conformation in determining the vibrational properties/signature patterns. In particular, the stretching mode at 1530–1560 cm^−1^, characteristic of the chemical bonds in the quinoidal resonance structure (see below for details), is found to be a sensitive indicator for chromophore–environment interactions and electron density redistribution upon light- or chemically induced structural changes. This work represents the first systematic vibrational characterization of FPs with different chromophore structures, environments, and conformations by use of a powerful tabletop vibrational spectroscopic technique with preresonance Raman enhancement to significantly improve the signal-to-noise ratio [26,54]. Our findings are anticipated to facilitate future mechanistic investigations and the targeted engineering of the structure and dynamics of diverse FPs for broad applications across the chemical, physical, and biological fields.

## 2. Results and Discussion

### 2.1. Effects of Sidechains in the GFP Model Chromophore

**Figure 2:**
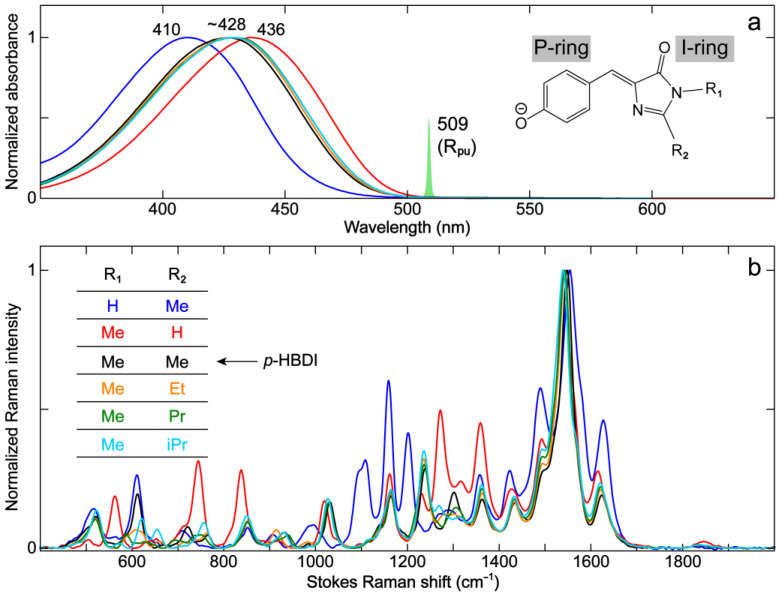
Steady-state electronic and vibrational characterization of GFP model chromophore derivatives. (**a**) Steady-state electronic absorption and (**b**) ground-state FSRS spectra of GFP model chromophores with different saturated substituents (color-coded) in aqueous solution. The spectral profile of the experimental Raman pump (R_pu_) pulse at 509 nm is scaled and shown in (**a**), with the chemical structure framework depicted in the inset. The P-ring and I-ring of the chromophore are labeled. The FSRS spectra are collected on the Stokes side with respect to the R_pu_ center wavelength. All the model chromophores are fully deprotonated in 10 mM NaOH aqueous solution.

Due to the relative simplicity in the treatment of the chromophore environment, the analysis of FP model chromophores in solution (e.g., with water solvent) is useful in understanding the chromophore properties inside the more heterogeneous protein matrix. As the model chromophore of GFP, *p*-HBI has been intensively investigated, with particular emphasis on its dimethyl derivative *p*-HBDI (R_1_ = –Me, R_2_ = –Me, Figure 1a) [28,29,30,55,56,57,58,59,60,61,62]. The model chromophore *p*-HBDI differs from the GFP chromophore in the substitutions R_1_ and R_2_ that are truncated with methyl groups, which are instead “hinge” points connected to the protein backbone [2,11]. To examine the effects of saturated R_1_ and R_2_ substituents, we incorporated –H and various alkyl groups such as –CH_3_ (–Me), –CH_2_CH_3_ (–Et), –CH_2_CH_2_CH_3_ (–Pr), and –CH(CH_3_)_2_ (isopropyl, –iPr) for the R_2_ substitution and –H and –Me for the R_1_ substitution (Figure 2). These synthetic chromophores are all deprotonated at the phenolic hydroxyl end in basic aqueous solutions due to the typically higher p*K*_a_ than the imidazolinone (I)-ring imine nitrogen (Figure 2a inset). For example, *p*-HBDI has a p*K*_a_ value of ~8.4 for the phenolic hydroxyl and ~2–3 for the I-ring imine nitrogen [61,63].

These anionic chromophores in water absorb in the 400–450 nm region. The R_2_ substitutions by alkyl groups (–Me, –Et, –Pr, and –iPr) lead to essentially no change in the electronic absorption peak wavelength (~428 nm, Figure 2a). This result is expected because the conjugation state is not changed. Interestingly, prominent shifts in the absorption band are observed when R_1_ or R_2_ is –H (blue or red trace in Figure 2). The R_2_ substitution by –H red-shifts the absorption peak (436 nm) compared to alkyl groups, which can be explained by the weak yet noticeable electron-donating capability of the alkyl groups. Previous studies have shown that R_2_ substitutions by electron-withdrawing groups (EWGs) promote the photoinduced intramolecular charge transfer (ICT) from the phenolic/phenolate (P)-ring to the I-ring (see labels in Figure 2a inset) and hence red-shift the chromophore electronic spectral peaks [64,65,66]. It is also consistent with the redder absorption/emission in RFPs than GFPs due to the enhanced ICT, which is induced by the extended conjugated moiety at the I-ring end [5]. In contrast, the R_1_ substitution by –H blue-shifts the absorption peak (410 nm) compared to –Me. The R_1_ substitution by an EWG has been previously shown to red-shift the absorption, which is less significant than the EWG at R_2_ [64]. Herein, the opposite trend by –H substitution at the R_1_ site likely arises from the strengthened chromophore–solvent interactions, such as the H-bonding between water and the specific NH moiety. Nevertheless, alkyl substitutions at the R_2_ site (with no conjugation to the chromophore aromatic system) do not perturb the electronic structure of *p*-HBI and thus minimally shift the chromophore’s absorption profile.

To further evaluate the effects of these substituents, ground-state FSRS spectra under preresonance conditions using a 509 nm narrowband R_pu_ pulse were collected (Figure 2, see Materials and Methods Section 3.3 for more details). Several observations can be made for the overlaid spectra in Figure 2b. (1) High-frequency modes in the 1400–1700 cm^−1^ region that mainly involve double-bond stretching motions remain largely unchanged with different alkyl substituents of R_2_. This result is in accord with their similar absorption wavelength, indicative of a negligible effect on the conjugated electronic structure. (2) The modes in the 1000–1400 cm^−1^ region, involving bending or rocking motions of the C–H bonds in the P-ring, methine bridge, and/or alkyl groups, do not shift with different alkyl substituents. (3) The modes below 1000 cm^−1^ mainly comprise chromophore skeletal motions, which involve the sidechains, and may change with different alkyl substituents.

**Figure 3:**
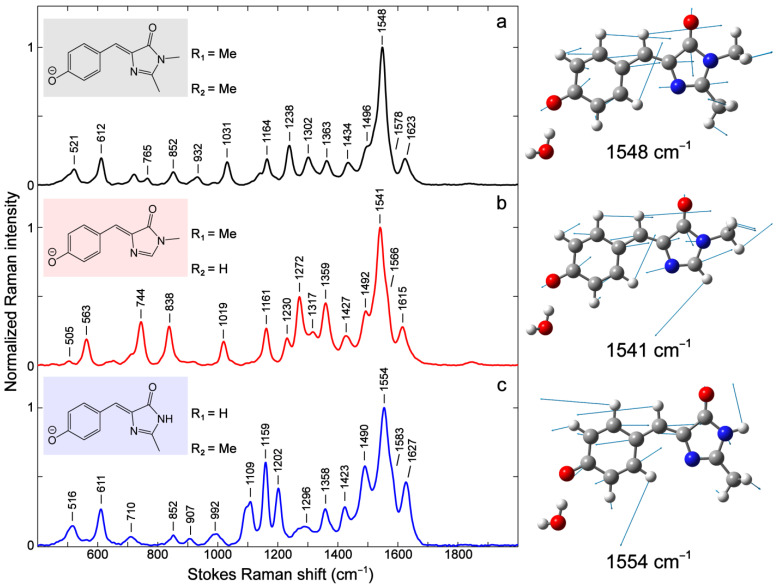
Effects of –H and –Me substituents on the vibrational modes of the GFP model chromophore. Ground-state FSRS spectra are collected on the Stokes side with respect to the 509 nm Raman pump. Most Raman peaks are labeled across the detection spectral window. All three model chromophores are deprotonated in 10 mM NaOH aqueous solution. The chemical structures with specific R_1_ and R_2_ substituents are depicted in panels (**a**–**c**) insets. The calculated vibrational motions with atomic displacement arrows of the most intense Raman mode are shown in right panels. Atom colors: C, gray; N, blue; O, red; and H, white. See Section 3 for details of the calculations and Table S1 for the mode assignment of these three contrasting GFP chromophore derivatives in water.

Notably, the R_1_ and R_2_ substitutions by –H lead to significant changes in mode intensity and frequency (see blue and red traces in Figure 2b) as well as mode compositions due to the absence of an alkyl group (see Figure S1a–c and Table S1 in the Supplementary Materials). The relative increase in mode intensity can be observed in the 700–900, 1100–1200, and 1250–1400 cm^−1^ regions and a few high-frequency modes above 1400 cm^−1^. Interestingly, the modes above 1400 cm^−1^ are shifted when an alkyl substituent like –Me is changed to –H. The strongest mode at 1548 cm^−1^ for *p*-HBDI (Figure 3a) red- and blue-shifts to 1541 and 1554 cm^−1^ when –Me is replaced with –H for R_2_ and R_1_, respectively. The trend seems to align with the electronic absorption peak wavelength, i.e., the mode frequency red-shifts with redder absorption (Figure 2). This marker band is assigned to P-ring quinoidal C=O/C=C, methine bridge C=C/C–C, and I-ring C=O/C=N stretching as well as the sidechain methyl C–H bending motions (or C–H/N–H rocking motions when substituted with –H, see Figure 3 right panels for the pertinent atomic displacements).

**Figure 4:**
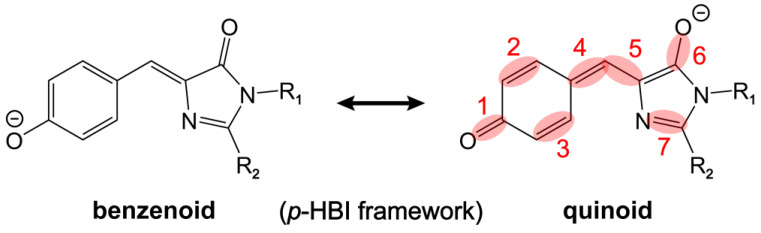
Two resonance structures of the anionic *p*-HBI chromophore. The bonds involved in the pronounced high-frequency stretching modes are shaded and numbered in red. Various R_1_ and R_2_ substituents of the *p*-HBI framework can be found in the inset of Figure 2b.

Using resonance theory, this quinoidal stretching mode is essentially indicative of a shift between the benzenoid and quinoid resonance structures of the chromophore (the stretching bonds are shaded in red, see Figure 4) [28]. First, the electron-withdrawing substitution of R_2_ promotes electron delocalization and results in a shift from the benzenoid to quinoid structure. The –Me group is less electron-withdrawing than –H, and the R_1_ = –Me/R_2_ = –H substitution should thus lead to a more quinoidal character with respect to R_1_ = –Me/R_2_ = –Me (i.e., the dimethyl derivative *p*-HBDI). This insight is supported by the bond length calculations (see Table 1) showing the shortened C=O/C=C (bonds 1, 2, 3, and 4 on the P-ring and methine bridge) and lengthened C–C/C=O (bonds 5 and 6 on the methine bridge and I-ring, Figure 4) from R_2_ = –Me to R_2_ = –H. We note that the R_1_ = –Me/R_2_ = –H substitution leads to a red-shifted electronic absorption peak with respect to R_1_ = –Me/R_2_ = –Me (Figure 2a). This result agrees with a recent report by Lin et al. on the color-tuning mechanism for GFP based upon a Marcus–Hush model which treats the anionic GFP chromophore as a superposition of the benzenoid and quinoid resonance structures [67]. It was proposed that the GFP color can be tuned and correlated with the driving force, i.e., the energy difference between the two resonance structures. The model suggests that stabilizing the charge on the I-ring by means of adding H-bonding partners and attaching EWGs can lower the energy of the quinoid structure (see Figure 4 right structure for the negative charge location) and thus red-shift the absorption due to the reduced driving force between the two resonance structures. Therefore, the redshift of the absorption peak by an electron-withdrawing R_2_ substitution [65,67] can be correlated with the more pronounced quinoidal character of the *p*-HBI chromophore (Figure 4).

Second, compared to the –Me substituent, the R_1_ substitution by –H leads to a very slight shift from the benzenoid to quinoid resonance structure which, however, drastically differs at the I-ring C=O bond from the R_2_ = –H substitution (Table 1). The calculated bond lengths show that the quinoidal bonds 1, 2, 3, and 4 in the presence of –H for R_1_ are slightly shortened, whereas bond 5 is marginally lengthened versus *p*-HBDI (Table 1). The trend displayed for R_1_ = –H is similar to that for R_2_ = –H, but the change in magnitude with respect to R_1_ = R_2_ = –Me is much smaller for the R_1_ = –H case, consistent with the aforementioned R_2_ site sensitivity to EWG groups [65,67]. The most striking difference is the shortened I-ring C=O bond length when R_1_ = –H (see the bolded number in Table 1), which is inconsistent with the trend predicted by these relatively simple resonance structures. This effect can be better verified by their associated infrared spectra because the I-ring C=O stretch has been shown to be a localized mode [30] with high sensitivity to its local environment [24,68].

Due to the significant involvement of the characteristic chemical bonds in the quinoid resonance structure (e.g., a clear trend for the collective bond length changes for bonds 1–4 in Table 1), one would expect a blueshift of this quinoidal stretching mode around 1541–1554 cm^−1^, and the mode frequency should follow the order of –H (R_2_) > –H (R_1_) > –Me (R_1_ and R_2_). Interestingly, it is counterintuitive that the observed mode frequency does not blue-shift when there is a more quinoidal character of the chromophore. Instead, the observed Raman mode frequency appears in the order of –H (R_1_) > –Me (R_1_ and R_2_) > –H (R_2_) (i.e., 1554 > 1548 >1541 cm^−1^, see Figure 3). The P-ring C=O/C=C and bridge C=C/C–C bonds do not seem to dictate the observed peak frequency shift. Instead, this marker band frequency blueshift/redshift is consistent with the shortening/lengthening of the I-ring C=O bond length (i.e., bond 6, see Figure 4 and Table 1). This key correlation indicates that the frequency shift induced by the bond length change (either lengthening or shortening) of this specific C=O bond likely overwhelms the shortening of the other quinoidal double bonds and therefore becomes the determining factor when –Me is switched to –H. As further evidence, the mode at 1623 cm^−1^ for *p*-HBDI involves similar stretching motions to the more intense 1548 cm^−1^ mode but less sidechain motions (i.e., R_1_ and R_2_) (see Figure 3a and Table S1). It exhibits the same frequency shift as the 1548 cm^−1^ mode when the sidechain –Me groups are separately replaced with –H in the order of –H (R_1_) > –Me (R_1_ and R_2_) > –H (R_2_) (i.e., 1627 > 1623 >1615 cm^−1^, Figure 3).

Through this systematic analysis of the contrasting *p*-HBI derivatives, it is clear that the high-frequency vibrational modes of GFP chromophores are highly sensitive to the I-ring sidechain modifications, even with substituents of a subtle difference in electronic properties such as –H and –Me. The quinoidal stretching modes are good indicators for electron delocalization which become useful in predicting the electronic absorption peak shift and other optical properties. However, the mode frequency may not be straightforward to interpret because these highly delocalized stretching modes involve both shortening and lengthening covalent bonds upon the population shift between the benzenoid and quinoid resonance structures of the chromophore (Figure 4).

### 2.2. Effects of the Chromophore Environment

#### 2.2.1. The Model GFP Chromophore in Various Solvents

**Figure 5:**
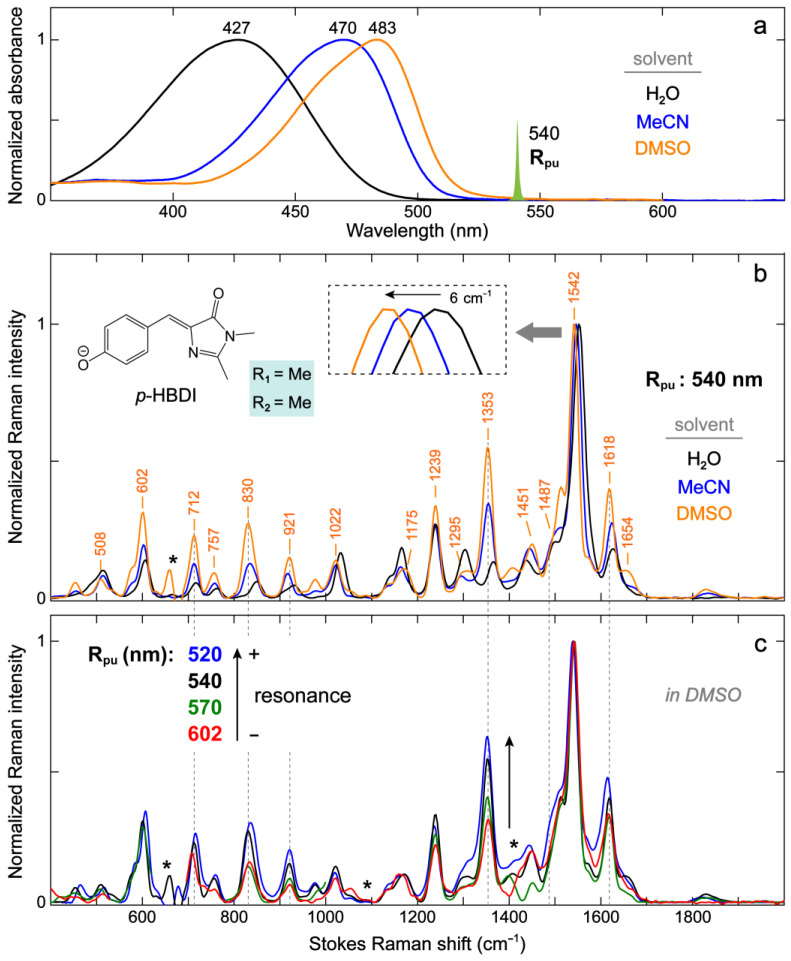
Ground-state (**a**) electronic absorption, (**b**) FSRS spectra of anionic *p*-HBDI in different solvents, and (**c**) FSRS spectra of anionic *p*-HBDI in DMSO with different R_pu_ wavelengths. The spectral profile of the R_pu_ pulse at 540 nm is scaled and shown in (**a**). The FSRS spectra are collected on the Stokes side with respect to R_pu_. The asterisks mark the solvent peak regions that interfere with the chromophore peaks. The chromophore is deprotonated in water by adding 10 mM NaOH and in MeCN and DMSO by adding 0.05–0.1% (*v*/*v*) DBU (1,8-diazabicyclo[5.4.0]undec-7-ene). The spectra are color-coded to represent *p*-HBDI in three solvents (**a**,**b**) and with four R_pu_ wavelengths (**c**). The notable peak frequency shift of the most intense marker band is magnified in the panel (**b**) inset. The prominent vibronically coupled modes are denoted by vertical dashed gray lines across panels (**b**,**c**).

The photophysical properties of the anionic GFP chromophores are known to be sensitive to their local environment, which usually involves H-bonding and dipole–dipole interactions. We selected protic (water) and aprotic (acetonitrile or MeCN and dimethyl sulfoxide or DMSO) solvents to examine these interactions for the anionic *p*-HBDI (i.e., R_1_ = –Me/R_2_ = –Me). The anionic *p*-HBDI exhibits pronounced solvatochromism, that is, the electronic absorption peak wavelength shifts with solvent polarity (Figure 5a). Previous Kamlet–Taft solvatochromic analysis has shown that the absorption energy gap of anionic *p*-HBDI in solvents is governed by both H-bonding and dipole–dipole interactions between the chromophore and solvent [69]. In particular, the H-bonding interaction increases the transition gap, while the dipole–dipole interaction shrinks it. This finding explains the bluer absorption in the protic water (427 nm), where H-bonding interactions are significant, and the redder absorption in non-H-bonding polar solvents like MeCN and DMSO (470 and 483 nm, Figure 5a). The solvatochromic analysis can be used to infer the ICT properties of the chromophore. The opposite effects on the electronic absorption energy by increasing the H-bonding and dipole–dipole interactions suggest that the H-bond-accepting capability and dipole moment of the anionic *p*-HBDI decreases and increases upon photoexcitation, respectively [65,70,71]. The weakened H-bonding capability of the chromophore in the excited state is reflective of the photoinduced ICT from the P-ring to I-ring which reduces the negative charge on the P-ring (i.e., –O^−^), which has been validated by many experimental and theoretical works [58,59,60,61,65,67,72,73].

In line with the solvent-dependent shift of the electronic absorption peak wavelength, the ground-state FSRS modes show unidirectional frequency shifts in the general order of water > MeCN > DMSO. For the vibrational modes above 1500 cm^−1^ that mainly involve double-bond stretching motions (see Figure S2 and Table S2 in the Supplementary Materials), the prominent peak frequency red-shifts from water (e.g., 1548 and 1623 cm^−1^, Figure 3a) to DMSO (1542 and 1618 cm^−1^, respectively, Figure 5b). Most modes below 1500 cm^−1^ also show similar redshifts (Figure 5b). The solvent-induced vibrational frequency shifts have been described by the vibrational Stark effect (VSE) for which the mode frequency red-shifts in a stronger electric field exerted by the solvent [74,75]. In previous VSE studies, water has usually been considered to produce a stronger average solvent electric field than DMSO despite its smaller dipole moment. The IR stretch frequency shift of carbonyls (–C=O) has been shown to linearly correlate to the average solvent electric field. For comparison, the linear relationship for a nitrile (–C≡N) frequency shift typically breaks down for protic solvents like water due to the H-bonding effect that causes a frequency blueshift [76,77]. More complicated relationships can also occur, as is the case for a deuterated aldehyde C–D stretch which blue-shifts and also linearly correlates with the average solvent electric field (i.e., positive electric field along the C–D bond) but with the opposite electric field magnitude to the carbonyl (i.e., negative electric field along the C=O bond, hence showing a frequency redshift as the solvent polarity increases) [78].

The observation that many modes of the anionic *p*-HBDI uniformly exhibit bluer frequencies in water than in DMSO makes it challenging to simply use the VSE terminology to interpret the solvent-dependent frequency shift. This trend might be caused and complicated by multisite solute–solvent interactions (H-bonding and/or dipole–dipole) that could lead to opposite effects on the bonds involved in the vibrational modes. For example, an H-bond between the chromophore phenolate group and a nearby water molecule tends to inhibit the delocalization of a negative charge across the aromatic ring system, producing a structure with more benzenoid character (Figure 4). The I-ring carbonyl has thus more double-bond character and could blue-shift the mode frequency. However, an H-bond between the I-ring carbonyl and an adjacent water molecule would red-shift the frequency (or produce a structure with more quinoid character, Figure 4) and promote more single-bond characters. Therefore, the apparent Raman mode frequency shift depends on the relative magnitudes of these specific interactions involving the chromophore.

This hypothesis was examined with the bond length calculations wherein an explicit water molecule was placed near the phenolate –O^−^ and/or I-ring C=O groups. When an explicit water molecule interacts with the phenolate –O^−^, the bonds 1, 2, 3, and 4 are lengthened while the bonds 5 and 6 are shortened when compared to the implicit solvation results (Table 2), reflecting a shift from the quinoid to benzenoid resonance structure (Figure 4). In contrast, these bond lengths exhibit opposite changes when an explicit water molecule was added to interact with the I-ring C=O (Table 2). The net effect (i.e., two explicit water molecules added with one interacting with the phenolate –O^−^ and the other with the I-ring C=O) seems to show that the H-bonding interaction between water and the phenolate –O^−^ outcompetes the one with the I-ring C=O, as suggested by the lengthening of bonds 1, 2, 3, and 4 and the shortening of bond 5. Interestingly, the I-ring C=O bond is still lengthened with respect to the implicit case, indicating the dominant role of a direct H-bond between water and the carbonyl groups. In fact, an H-bond between water and the phenolate –O^−^/I-ring C=O lengthens bond 1/bond 6 by ~0.01 Å (1.2823 vs. 1.2709 Å for bond 1 and 1.2462 vs. 1.2372 Å for bond 6) but changes other bond lengths by <0.005 Å (Table 2). This result suggests that the H-bonding effect is quite localized, and the shift between resonance structures represents a simplified treatment of the effects of chromophore–solvent interactions that requires a more sophisticated analysis and useful insights. Although we could glimpse the H-bonding effects through quantum chemical calculations with explicit solvent molecules, the resultant bond lengths (Table 2) remain inconsistent with the observed frequency blueshift of high-frequency modes from DMSO to water (e.g., 1542 to 1548 cm^−1^ and 1618 to 1623 cm^−1^, see Figure 3a and Figure 5b). This discrepancy might be due to the oversimplified treatment of H-bonding interactions with only a couple of explicit solvent molecules and/or the insufficiency of the current level of theory in our calculations. Higher-level calculations that describe the solvation better such as the condensed phase that includes a cluster of solvent molecules surrounding the chromophore, hybrid/long-range-corrected models, quantum mechanics/molecular mechanics (QM/MM), and ab initio molecular dynamics (AIMD) simulations are needed to shed more light on the solvent effects on a dynamic chromophore like *p*-HBDI [62,79,80,81,82,83].

Besides the peak frequency shift, the Raman mode intensity also demonstrates notable solvent-dependent changes. As the electronic absorption peak of anionic *p*-HBDI red-shifts from water to MeCN to DMSO, several modes with the same R_pu_ wavelength (540 nm) exhibit an intensity increase at ~712, 830, 921, 1353, 1487, and 1618 cm^−1^ relative to the most intense mode at 1542 cm^−1^ (in DMSO, Figure 5b). This trend can be understood by Albrecht’s theory on resonance Raman intensities which states that the Raman vibrations coupled to the electronic transition (i.e., vibronic coupling) should gain intensity relative to all other vibrations as resonance is approached [84,85]. It is noted that as the electronic absorption of anionic *p*-HBDI red-shifts, the dual-band vibronic coupling feature becomes more apparent (Figure 5a). We thus used the second derivatives of the absorption spectra to estimate the vibronically coupled modes and found a dominant mode at ~1230–1350 cm^−1^ in organic solvents (see Figure S3 in the Supplementary Materials). The frequency closely matches the one at 1353 cm^−1^ that exhibits a significantly larger intensity in DMSO than water (Figure 5b), indicating that this specific mode is strongly coupled to the electronic ground-to-excited state transition. For corroboration, we tuned the R_pu_ wavelength across an 80+ nm region and compared the relative mode intensities of anionic *p*-HBDI in DMSO (Figure 5c). The relative peak intensity increase in the 1353 cm^−1^ marker band as the resonance condition is enhanced stepwise from 602 to 520 nm confirms the vibronic coupling effect on Raman mode intensities from the ground-state FSRS measurements [31,85]. A few other modes at ~712, 830, 921, 1487, and 1618 cm^−1^ (across the detection window, see dashed gray lines in Figure 5b,c) also demonstrate relative intensity increases as the R_pu_ is tuned from the red side (e.g., 602 nm) toward the electronic absorption peak at 483 nm (Figure 5a). These vibrational modes are consistent with those that exhibit the peak intensity increases from water to MeCN and then DMSO (Figure 5b,c), involving characteristic nuclear motions (see Tables S1 and S2 in the Supplementary Materials) with relatively large displacements from S_0_ to S_1_, thereby highlighting the role of vibronic coupling in resonance Raman intensities of GFP model chromophores in solution.

#### 2.2.2. GFP Chromophores in Various Protein Matrices

**Figure 6:**
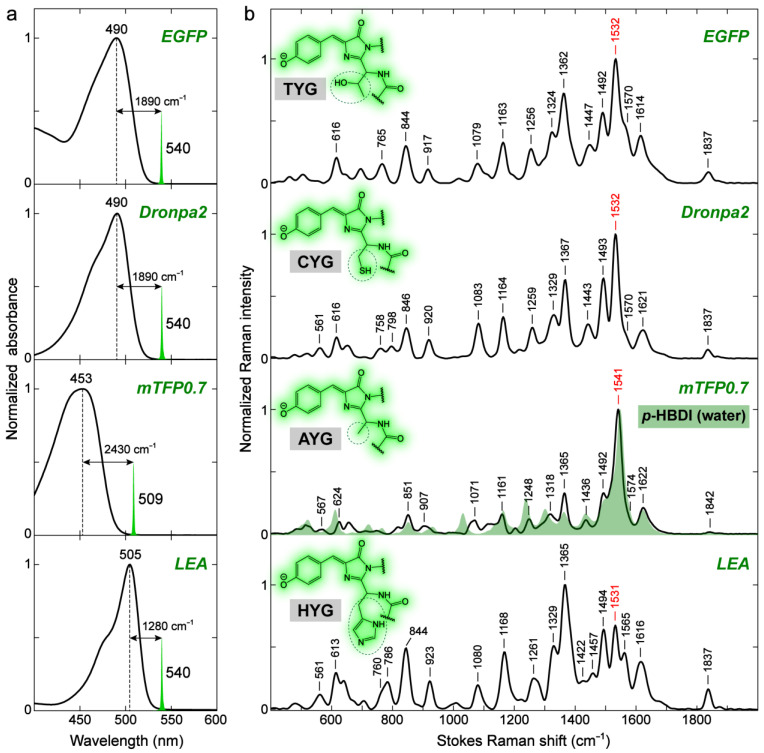
Ground-state (**a**) electronic absorption and (**b**) FSRS spectra of different GFPs in an aqueous buffer. The spectral profiles (green) of tunable R_pu_ at 540 nm for EGFP, Dronpa2, and LEA, and 509 nm for mTFP0.7 are scaled and shown in (**a**). The energy gap in the cm^−1^ unit between the absorption peak maximum and R_pu_ locations are labeled to indicate the resonance condition achieved in the experiments. The FSRS spectra are collected on the Stokes side with respect to R_pu_. The GFP chromophore moieties and pertinent amino-acid-abbreviated tripeptides are shown with green shades and gray boxes, respectively, in panel (**b**) insets. The distinct chromophore sidechains are highlighted by dashed ellipses. The spectrum of anionic *p*-HBDI in water (509 nm R_pu_, also displayed in Figure 3a) shown in a green shade is overlaid with mTFP0.7 for direct comparison. The peak frequency of a key vibrational marker band is highlighted in red (see main text for discussions).

**Figure 7:**
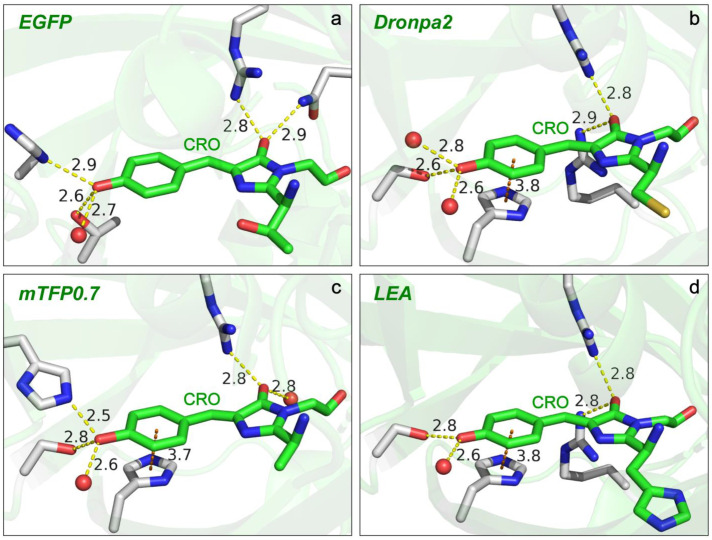
Local environment of the chromophores in different GFPs revealed by crystal structures. The associated Protein Data Bank (PDB) IDs are (**a**) 4EUL (EGFP), (**b**) 6NQJ (Dronpa2), (**c**) 2OTB (mTFP0.7), and (**d**) 4DXN (LEA) (see main text for the supporting literature).The H-bonds and π–π-stacking interactions between the chromophore (CRO, carbon atoms denoted in green) and surrounding protein residues (carbon atoms denoted in gray) or water molecules (oxygen atom denoted as a red sphere) are indicated by yellow and orange dashed lines, respectively, with the pertinent atomic distances (numbers with two significant digits) labeled in the angstrom (Å) unit. Nitrogen atoms are shown in blue, and hydrogen atoms are not shown. The semi-transparent green ribbons in the background depict the surrounding β-barrel structure.

Compared to solvents, the chromophore environment in FPs is more heterogeneous and much more challenging to describe in a highly precise manner. The Marcus–Hush model of Lin et al. [67] provides a convenient approach to understand the correlation between the protein environment and the photophysical properties of GFP, such as the electronic absorption wavelength, Stokes shift, vibronic coupling, etc. Specifically, for the electronic absorption wavelength, a rule of thumb based on the model is that any interactions that stabilize the negative charge at the P-ring (benzenoid structure) or destabilize the negative charge at the I-ring (quinoid structure, Figure 4) will blue-shift the absorption peak. Due to the fact that strong interactions such as H-bonding with the chromophore in GFPs often occur at the sites of the P-ring –O^−^ and I-ring carbonyl, one can thus generally understand the absorption blue-/redshift in GFPs by inspecting the difference of the chromophore–environment interactions at these two sites, reminiscent of the aforementioned discussions for *p*-HBDI in solution. Such site-specific interactions are also expected to affect the vibrational properties of the chromophore inside GFPs. We strategically selected four different GFPs (i.e., EGFP, Dronpa2, mTFP0.7, and LEA green form) that absorb at relatively separated wavelengths (see Figure 6a and Figure S4a) to examine and characterize the environmental effect via ground-state FSRS (see Figure 6b and Figure S4b,c for more details). Dronpa2 and mTFP0.7 are photoswitchable FPs, and the resting “on” state with a *cis*-anionic chromophore was studied for relevant comparisons [86,87,88]. LEA has been recently found to be both photoswitchable and photoconvertible, and similarly, the unconverted “on” state with a *cis*-anionic chromophore was compared in the literature [10,51,89]. Using the original and popular EGFP as a reference (Figure 7a) [90], Dronpa2/mTFP0.7/LEA absorbs at a similar/bluer/redder wavelength, respectively (Figure 6a), which reflects the similarity or difference in their embedded chromophores’ local environments, as depicted in Figure 7b–d.

The electronic absorption peak shift can be rationalized by the difference in the number and/or strength of the charge-stabilizing (mainly through H-bonding) partners near the chromophore [67]. Compared to Dronpa2 [88], illustrated in Figure 7b, the green form of LEA [10] has less H-bonding partners (water) and a longer H-bond at the P-ring end, but shorter H-bonds with two arginine residues at the I-ring end (see crystal structure in Figure 7d) [10]. The negative charge is thus less stabilized at the P-ring but more stabilized at the I-ring in LEA, both of which contribute to a redshift in electronic peak absorption according to Lin’s model [67] (see Figure 6a, bottom panel). Likewise, the significantly blue-shifted absorption of mTFP0.7 (Figure 6a, second-to-bottom panel) can be ascribed to the absence of a strong H-bonding donor, arginine, at the I-ring and the presence of a strong H-bond between an adjacent histidine and the chromophore P-ring phenolate end [87]. The more stabilization at the P-ring and less stabilization at the I-ring additively lead to a notably blue-shifted absorption peak in mTFP0.7 with respect to the other three GFPs. Furthermore, the similar electronic absorption for EGFP and Dronpa2 (see the top two panels of Figure 6a for the absorption spectra and Figure 7a,b for the crystal structures) likely arises from a cancellation effect, i.e., less stabilization at the I-ring (with a weaker H-bond partner, glutamine, than arginine) in EGFP is compensated by less stabilization at the P-ring (weaker H-bonding interactions due to more distant partners).

The preresonance ground-state FSRS spectra with high signal-to-noise ratios and absorptive line shapes (see Figure S4b,c for the spectra of EGFP with R_pu_ wavelengths from off-, pre- to on-resonance conditions) were collected to shed crucial light on the chromophore–environment interactions with chemical bond precision (Figure 6b). In terms of the observed Raman peak frequencies, the three redder GFPs (EGFP, Dronpa2, and LEA) manifest similarities for modes across a broad spectral range, with only small shifts within ~5 cm^−1^. In contrast, the blue-absorbing mTFP0.7 demonstrates notable frequency differences as large as 10–16 cm^−1^ from the three GFPs for many modes (see Figure 6b and Table 3). Interestingly, the Raman peak frequencies are not unidirectionally shifted but exhibit mode-dependent blueshifts or redshifts. The high-frequency modes above 1500 cm^−1^, characteristic of double-bond stretching vibrations (see Section 2.1 and Section 2.2.1), are generally blue-shifted from those of the other three GFPs. These bond-stretching frequency shifts in GFPs can be understood in the same manner as for the model chromophore *p*-HBDI in solvents. The H-bonds with the P-ring –O^−^ and I-ring carbonyl push the resonance structure toward benzenoid and quinoid, respectively (Figure 4), which can shift the vibrational mode frequency in opposite directions and support its use as a sensitive marker band inside the protein pocket.

In comparison to EGFP, mTFP0.7/LEA has stronger/weaker and weaker/stronger H-bonding interactions with the P-ring –O^−^ and I-ring carbonyl, respectively (Figure 7) [10,87,90]. The chromophore of mTFP0.7 is thus shifted toward the benzenoid structure, while that of LEA has a more quinoid character. As mentioned above (also see Table 1), the relative frequency shifts of modes above 1500 cm^−1^, such as the most intense one around 1500–1550 cm^−1^, are effectively affected by the bond length change in the I-ring carbonyl group despite the delocalized motions of these vibrational modes. This key insight is further substantiated by the frequency blueshift and redshift in mTFP0.7 and LEA (Figure 6b) due to the more pronounced benzenoid and quinoid character, respectively. Dronpa2 is highly similar to EGFP in mode frequency likely due to a cancellation effect as also manifested by their identical electronic absorption peak wavelengths (Figure 6a, top two panels).

Notably, mTFP0.7 resembles *p*-HBDI in water in mode frequency the most, mainly in the region above ~1350 cm^−1^, with respect to the other three redder GFPs (see the overlaid spectra of mTFP0.7 and *p*-HBDI in water, Figure 6b). To be exact, *p*-HBDI peaks are still slightly bluer than mTFP0.7 for these high-frequency stretching modes (Table 3). This observation is indicative of the H-bonding environment for the model GFP chromophore in water, i.e., the H-bonding interaction is much stronger at the P-ring –O^−^ end than at the I-ring carbonyl end. It may also explain the further blue-shifted electronic absorption peak for the model chromophore *p*-HBDI in water versus mTFP0.7 (427 nm in Figure 5a vs. 453 nm in Figure 6a). The interplay between the VSE-induced mode frequency redshift or blueshift [78] and the electronic color-changing effects (e.g., benzenoid and quinoid forms in Figure 4) via H-bonding interactions could lead to the observed blueshift in bond-stretching frequencies from aprotic to protic solvents (e.g., DMSO to water, Figure 5b).

Another drastic difference in the vibrational modes of various GFPs is the mode intensity, which can be attributed to the resonance enhancement for vibronically coupled modes according to Albrecht’s resonance Raman theory [84,85,91]. Among the four GFPs with similar green chromophore structures studied in this work, mTFP0.7 absorbs at the bluest wavelength and has similar relative mode intensities to the anionic *p*-HBDI in water (Figure 6b, second-to-bottom panel). As the electronic absorption peak red-shifts from mTFP0.7 to EGFP/Dronpa2 and then to LEA, a number of modes demonstrate the intensity increase relative to the dominant mode at 1541 cm^−1^ (in mTFP0.7) or its counterpart in the other GFPs, which include all the Raman modes labeled in Figure 6b and listed in Table 3. These modes are similar to those observed for *p*-HBDI in DMSO (Figure 5c), suggesting that they are to some extent vibronically coupled to the electronic absorption, especially for the redder-absorbing GFPs. In particular, the ~1365 cm^−1^ mode shows a pronounced intensity increase as the electronic absorption peak red-shifts (Figure 6a) and becomes the most intense mode in LEA, which agrees with the second-derivative analysis that reveals a strongly coupled vibration at ~1357 cm^−1^ (see Figure S4a in the Supplementary Materials). Further insights into the functional role of this mainly bridge and P-ring H-rocking motion upon photoexcitation and out of the Franck–Condon region could inspire more advanced experimental and computational studies [26,62,85,92,93].

### 2.3. Effects of the Chromophore Structure

#### 2.3.1. Yellow, Orange, and Red Fluorescent Protein (YFP, OFP, and RFP)

**Figure 8:**
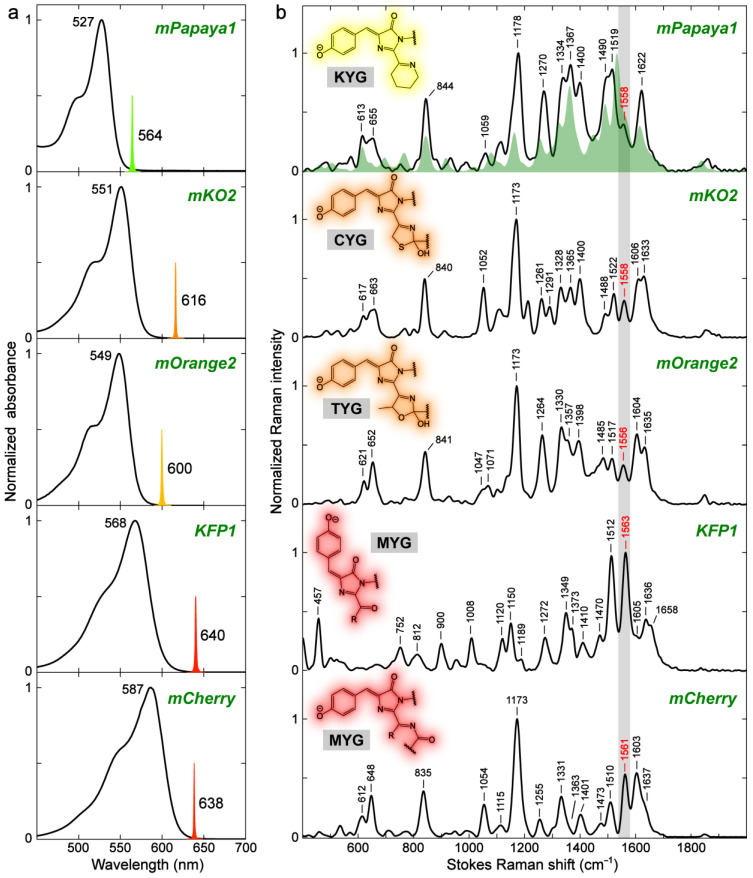
Ground-state (**a**) electronic absorption and (**b**) FSRS spectra of various FPs with the conjugation-extended chromophores in an aqueous buffer. The Raman pump (R_pu_) spectral profiles at 564 nm for mPapaya1, 616 nm for mKO2, 600 nm for mOrange2, 640 nm for KFP1, and 638 nm for mCherry are scaled and shown in (**a**) with coded colors. The FSRS spectra were collected on the Stokes side with respect to the R_pu_ center wavelength. The FP chromophore moieties and pertinent amino-acid-abbreviated tripeptides are shown with the color-coded shades and gray boxes, respectively. The EGFP spectrum (same as the one in Figure 6b) shown in a green shade is overlaid with mPapaya1 for comparison. Note that the chromophore in mOrange2 was not fully matured and there is a portion of unmatured green chromophore population. See Figure S5 for the decomposed electronic absorption spectra of the matured and unmatured chromophores. Nevertheless, the FSRS spectrum with 600 nm R_pu_ primarily captures the matured orange chromophore by resonance enhancement, as corroborated by similar mode frequencies to mKO2 which has the same conjugation structure with subtle differences in the I-ring sidechain heterocycle (see the orange-shaded chemical structures in the insets of panel **b**). The peak frequency of a key vibrational marker band is highlighted in red color and by a thin rectangular box in gray color (see main text for discussions).

For a comprehensive comparison of the FPs beyond GFP, we expressed mPapaya1, mKO2, mOrange2, KFP1, and mCherry as representative proteins that consist of each chromophore structure (Figure 1). Due to the difference in conjugation size and electron-withdrawing strength of an extended moiety, mPapaya1 and mKO2/mOrange2 fall into the yellow and orange emission regions, respectively, while KFP1 and mCherry are RFPs (Figure 8a). The low chromophore p*K*_a_ values result in the predominantly deprotonated (anionic) chromophores in these FPs. Notably, KFP1 is a photoswitchable RFP mutated from asFP595 and the resting off-state (i.e., very low FQY) has a *trans*-anionic chromophore [49,94].

The preresonance (see tunable R_pu_ positions in Figure 8a) FSRS spectra were collected to obtain high signal-to-noise ratios for these colorful FPs in the visible spectral region (Figure 8b). Surprisingly, mPapaya1, mKO2, mOrange2, and mCherry demonstrate high spectral similarity in mode frequencies despite the variations in their chromophore structures which, however, have a common imine (–C=N–) moiety connected to the I-ring end. mCherry shows relatively large frequency shifts from mPapaya1/mKO2/mOrange2. For example, the 1473 and 1510 cm^−1^ modes of mCherry are red-shifted by 7–17 cm^−1^ from the other FPs, which likely reflect an extended conjugation due to the –C=N–C=O chain in mCherry (Figure 8b, bottom panel). The systematic quantum calculations show that many vibrational normal modes have conserved motions (see Figure S6 and Table S3 in the Supplementary Materials) across different FPs. In contrast, KFP1, whose chromophore has a different double bond (i.e., –C=O) directly connecting to the I-ring R_2_ site, exhibits substantial changes in the motions of normal modes and frequency shifts for the same or similar normal modes as the other four FPs (see Figure 8b, Figure S6, and Table S4 in the Supplementary Materials). In particular, the absence of a peak doublet between ~600 and 700 cm^−1^ as well as the altered intensity ratio of the ~1560 cm^−1^ mode over 1150 cm^−1^ mode in KFP1 are conspicuous (Figure 8b). It seems that the vibrational frequencies are significantly related to the structure of conjugated groups, largely regardless of the nonconjugated saturated moieties [95]. For rigorous analysis, the conformational effect due to the *trans* chromophore in KFP1 needs to be taken into consideration (see below).

Compared to GFPs, the redder FPs with different chromophore structures (more substantial than the –H/–Me sidechains in Section 2.1) show considerable changes in normal modes due to the involvement of nuclear motions from the extended moieties that play a role in electronic conjugation (see Tables S1 and S3 in the Supplementary Materials). We also note that some vibrational normal modes are largely conserved between GFPs (Section 2.2.2) and these Y/O/RFPs. For example, the most intense mode in EGFP at ~1532 cm^−1^ due to the quinoidal stretch (Figure 4 and Figure 6b) is retained in its vibrational motions but blue-shifted in mPapaya1 (1558 cm^−1^), mKO2 (1558 cm^−1^), mOrange2 (1556 cm^−1^), KFP1 (1563 cm^−1^), and mCherry (1561 cm^−1^, Figure 8b). The rather large blueshift (~20–30 cm^−1^) may not be solely explainable by the environmental effect [24,28,95]. Instead, it is likely caused by the EWGs (–C=N, –C=O, or –C=N–C=O) at the I-ring end that can induce a different electron density on the quinoidal stretching bonds (Figure 4).

**Figure 9:**
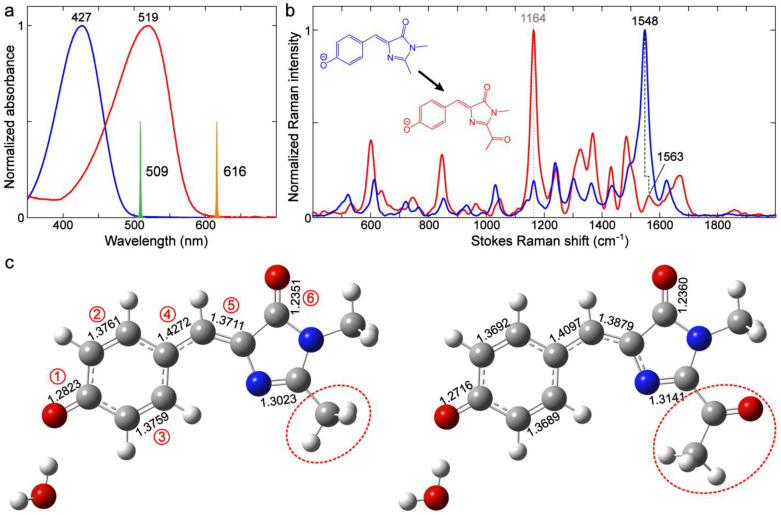
Comparison of ground-state (**a**) electronic absorption, (**b**) FSRS spectra, and (**c**) calculated bond lengths between *p*-HBDI (blue) and the *cis* isomer of the KFP1 model chromophore (red) in water. The Raman pump (R_pu_) spectral profiles at 509 nm for *p*-HBDI and 616 nm for the KFP1 model chromophore are scaled and shown in (**a**) as color-coded spikes. The FSRS spectra were collected on the Stokes side with respect to the R_pu_ center wavelength. Both model chromophores are deprotonated (anionic state) in 1–10 mM NaOH aqueous solution. The frequency blueshift of the characteristic quinoidal stretching mode is indicated by black dashed lines. The most prominent peak for the *cis* KFP1 model chromophore is denoted by the gray dotted line. See Section 3.4 for the calculation details. In panel (**c**), the characteristic bond lengths (see Figure 4 for the bond numbering and definition of the two resonance structures) are labeled, while distinct sidechains on the I-ring R_2_ site are enclosed by red dashed ellipses. Atom colors: C, gray; N, blue; O, red; and H, white.

The environment-induced frequency shifts in FPs (e.g., see Figure 6 and Figure 7) may complicate those caused by variations in the chromophore structure, thus making the interpretation less definitive. For deeper insights, we next turn to the model chromophores in various solvents to investigate the structural effect on the Raman frequency shifts. Unfortunately, the model chromophores for most of these Y/O/RFPs have not been successfully synthesized outside the protein matrices. One exception is the model chromophore of KFP1 with an acetyl group (–COMe), which differs from the KFP1 chromophore (*trans* isomer, see Figure 8b inset in the second-to-bottom panel) in the conformation (see the *cis* isomer in Figure 9) [96,97,98]. While the *cis* form of the KFP1 model chromophore shows a red-shifted electronic absorption peak versus *p*-HBDI (Figure 9a) due to the EWG (–COMe) at the I-ring R_2_ site for extended conjugation [65], the quinoidal stretching mode (highlighted in Figure S7 in the Supplementary Materials) exhibits a 15 cm^−1^ blueshift (i.e., 1548 to 1563 cm^−1^, see Figure 9a,b).

We calculated the bond lengths for the two chromophores in water to rationalize the blueshift. As shown in Figure 9c, with one explicit water molecule at the P-ring end (same as Figure S1d, see Section 3.4 below for computational details), bonds 1, 2, 3, and 4 are shortened, whereas bonds 5 and 6 are lengthened, when the –CH_3_ group is replaced with –COMe. These bond length changes are characteristic of the shift from a benzenoid to quinoid resonance structure (Figure 4). Notably, the magnitude for the shortening of the P-ring and bridge bonds 1, 2, 3, and 4 (0.007–0.017 Å) is quite large, while the I-ring C=O bond length stays largely unchanged (from 1.2351 to 1.2360 Å, Figure 9c). The length changes in the quinoidal double bonds undoubtedly dictate the observed mode frequency blueshift in this case. Therefore, the large frequency blueshift for this quinoidal stretching mode from GFPs (Figure 6b) to Y/O/RFPs (Figure 8b) can be mainly attributed to the charge redistribution from the P-ring to I-ring, induced by the conjugated electron-withdrawing moiety at the I-ring end.

There seem to be discrepancies in the frequency shift trend between –Me and EWG (Figure 9) and between –Me and –H substitutions at the R_2_ site (Figure 2 and Figure 3), as well as for *p*-HBDI in various solvents (Figure 5). In the latter cases (Figure 2, Figure 3 and Figure 5), the quinoidal stretching mode exhibits a redshift with a red-shifted electronic absorption peak, reflecting a shift from a benzenoid to quinoid structure, and the calculations suggest this specific mode frequency to be effectively governed by the bond length change in the I-ring C=O group (Table 1). This point is corroborated by the similar mode frequency redshift in GFPs as the electronic absorption peak red-shifts due to altered H-bonding interactions at the I-ring and/or P-ring ends (Figure 6 and Figure 7). In contrast, an EWG with substantial strength such as –COMe at the I-ring R_2_ site results in a red-shifted absorption peak but a vibrational (quinoidal stretching) mode frequency blueshift (Figure 9b), which showcases the site-specific engineering potential of the versatile *p*-HBDI framework with rich photophysical insights. In particular, the relatively large magnitudes of such electronic absorption peak and vibrational frequency shifts induced by a strong EWG (Figure 9a,b) substantiate the general use of resonance structures in describing the properties of FP chromophores.

Notably, we have focused on the Raman mode frequency comparative analysis to gain molecular insights, aided by the calculated vibrational normal mode frequency pattern across various chromophore structures. On the other hand, the observed Raman mode intensity is complicated to fully analyze due to the vibronic coupling effect (see Section 2.2.1 and Section 2.2.2) and the intrinsic mode-dependent Raman activity (electric polarizability) for different chromophore structures [31,54,85,91]. A conspicuous difference among the aforementioned Y/O/RFPs in Raman mode intensity pattern (Figure 8b) is that the mode at ~1170–1180 cm^−1^ is the most intense peak for mPapaya1/mKO2/mOrange2/mCherry with a *cis*-anionic chromophore, which becomes much weaker (besides the shifted frequencies, see Table S4) in KFP1 with a *trans*-anionic chromophore (Figure 8b insets). For corroboration, the ~1164 cm^−1^ mode is the strongest peak for the *cis* isomer of the KFP1 model chromophore (Figure 9b), which is in accord with the predominantly *cis* conformation for GFP-like model chromophores including *p*-HBDI in solution [56]. Since this specific mode mainly involves the chromophore P-ring H-rocking motions (Tables S1–S4) [24,26,29,99] which are expected to probe the P-ring’s local environment, the diminishment of this mode could be used as a signature for the *trans* conformer during a light-induced or chemically induced reaction for such FPs.

#### 2.3.2. Kaede-Like RFP

**Figure 10:**
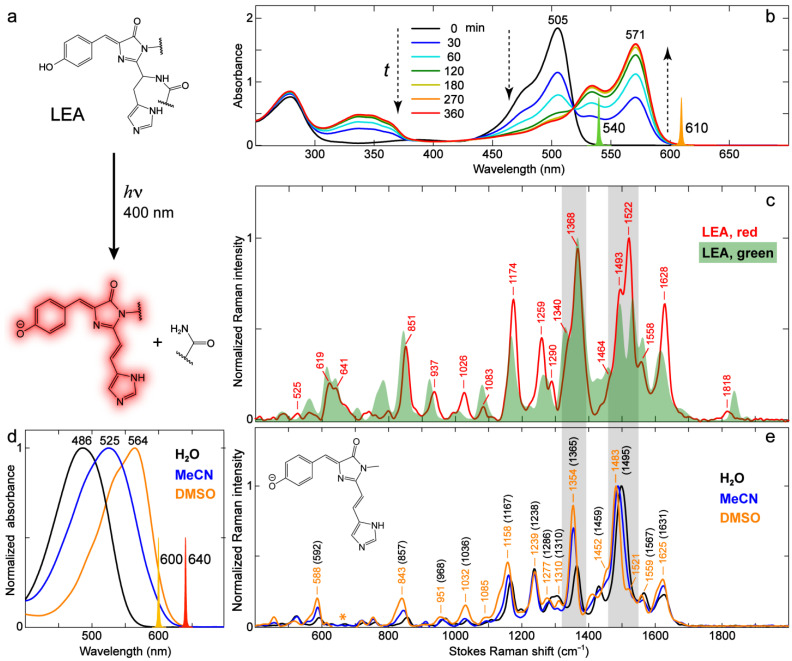
Characterization of the green-to-red photoconvertible LEA and its model red chromophore in various solvents. (**a**) Green-to-red photoconversion by 400 nm light irradiation. (**b**) Steady-state electronic absorption spectra of LEA upon 400 nm LED irradiation with time points color-coded and listed in the inset. Vertical dashed arrows denote the progression of time. The Raman pump (R_pu_) spectral profiles at 540 nm for LEA and 610 nm for the photoconverted LEA are scaled and shown as color-coded spikes. (**c**) Ground-state FSRS spectra of LEA (green form) and photoconverted LEA (red form) after 6 h of 400 nm LED irradiation. (**d**) Steady-state electronic absorption spectra of the deprotonated Kaede model chromophore (reminiscent of the photoconverted LEA red form) in three different solvents. The R_pu_ spectral profiles at 600 nm in water and 640 nm in MeCN and DMSO are scaled and shown as color-coded spikes. (**e**) Ground-state FSRS spectra of the deprotonated Kaede model chromophore in H_2_O (black), MeCN (blue), and DMSO (orange). All FSRS spectra are collected on the Stokes side with respect to the R_pu_ center wavelength. The FSRS spectrum of unconverted LEA (green form, Figure 6b) is shown in a green shade in (**c**). The orange asterisk marks the region of DMSO solvent peaks that interfere with the chromophore peaks in (**e**). The model chromophore is deprotonated in water by adding 1 mM NaOH, as well as in MeCN and DMSO by adding 0.05–0.1% (*v*/*v*) DBU. Key Raman marker bands are highlighted by two gray rectangular boxes across panels (**c**,**e**) with bold labels.

Besides RFPs with autocatalytically formed chromophores, Kaede-type FPs represent another class of RFPs that produces a red-emitting chromophore via photoconversion [8,9,10]. We selected an engineered least-evolved ancestor (LEA) protein that acquires green-to-red photoconversion capability [10,13] to investigate the vibrational properties of the photoconverted red chromophore. Figure 10a illustrates the putative chromophore structural change via photoconversion, wherein the neutral chromophore with a *p*-HBDI core is converted by 400 nm light irradiation to the red anionic chromophore with an extended conjugation [10,51,89] in concert with the ejection of a leaving group. We converted LEA with a 400 nm LED light, and the main absorption peak is gradually shifted from 505 nm (green form, anionic) to 571 nm (red form, anionic, see Figure 10b). The conversion seems to be complete after 360 min LED irradiation, as verified by the nearly identical profiles between the excitation and absorption spectra (Figure S8a) and the marginal green emission when the converted LEA is excited at 490 nm (Figure S8b).

The photoinduced structural change in the chromophore can also be captured by FSRS. The converted LEA shows significant spectral changes particularly in the high-frequency region above ~1400 cm^−1^ (Figure 10c). For a useful comparison, we synthesized the model chromophore of photoconverted LEA (referred to as the Kaede chromophore [5,8] hereafter) and measured its electronic absorption (Figure 10d) and preresonance FSRS (Figure 10e) spectra in different solvents (water, MeCN, and DMSO). Similar to *p*-HBDI in solution, the anionic Kaede chromophore exhibits pronounced solvatochromism in electronic absorption with a peak redshift from water to MeCN to DMSO (Figure 10d); we thus implemented the 600 and 640 nm Raman pumps to enhance the chromophore signal strength in water and MeCN/DMSO, respectively. We note that several prominent modes (e.g., 843, 1158, 1354, and 1483 cm^−1^, see peak labels in orange, Figure 10e) demonstrate marked frequency redshifts of ~10–20 cm^−1^ from water to DMSO, and the shift magnitudes are significantly greater than those of *p*-HBDI in water and DMSO (Figure 5b). The modes above 1400 cm^−1^ mainly involve bond-stretching motions, and their sensitivity to solvent polarity suggests that the electron density distribution of the Kaede chromophore is highly susceptible to the environment, which is in accord with its much more extended sidechain at the I-ring end (see the contrasting chemical structures in the inset of Figure 10e vs. the inset of Figure 5b). A similar analysis of frequency shift using the resonance structures is not readily applicable for the Kaede chromophore because the presence of the conjugated styryl imidazole group drastically changes the normal modes with respect to *p*-HBDI (see Figure S9 and Table S5 in the Supplementary Materials). Nevertheless, the generally blue-shifted vibrational peak frequency with a blue-shifted electronic absorption peak (e.g., 1495 and 1567 cm^−1^ in water vs. 1483 and 1559 cm^−1^ in DMSO) still matches the aforementioned trend (Figure 2, Figure 3, Figure 5 and Figure 6).

The relative mode intensity variation in the Kaede model chromophore between solvents can be generally explained by the vibronic coupling effect. In reference to the most intense mode (i.e., 1483 cm^−1^ in DMSO or 1495 cm^−1^ in water, Figure 10e), the 1350–1370 cm^−1^ mode exhibits the most pronounced intensity increase as the electronic absorption peak red-shifts from water to DMSO (Figure 10d). This interesting result is similar to *p*-HBDI from solvent water to DMSO (Figure 5b) and indicates that this specific vibrational mode is strongly coupled to electronic transition, which is consistent with its composition of the bridge H-rocking motions (see Tables S2 and S5) that have been shown to be highly sensitive to photoexcitation and the pertinent charge transfer across the chromophore framework [26,31]. This finding is further supported by the second-derivative analysis of the electronic absorption spectra for various samples, revealing a vibronically coupled mode at a similar frequency of ~1300 cm^−1^ (Figure S10 in the Supplementary Materials).

The photoconverted LEA demonstrates the mode-dependent resemblance in frequency to the red model chromophore in solution. For example, the modes at ~937 and 1558 cm^−1^ are closer in frequency to their counterpart modes of the deprotonated Kaede model chromophore in DMSO (Figure 10c,e). In contrast, many other modes (e.g., 1174, 1290, 1368, 1493 cm^−1^) of the photoconverted LEA have similar frequencies (as well as relative intensity ratios) to those of the model chromophore in water. This observation is similar to the comparison between GFPs and *p*-HBDI in solution which exhibits the mode dependence as well (Table 3). It is clear that the environmentally induced vibrational frequency shifts for this chromophore, particularly in the protein matrix, cannot be simply explained by a unified model such as the one proposed to correlate the GFP electronic absorption peak wavelength to the environment [67]. Considering the heterogeneous chromophore–environment interactions, such as the specific H-bonding interactions in FPs, the analysis of vibrational mode frequency should be on a mode-by-mode basis. In addition, the delocalized nature of many Raman modes makes it more challenging to fully interpret the mode frequency shift across a wide spectral window (Tables S1–S5).

Besides the influence of the protein environment on vibrational mode frequency, the mode intensities of photoconverted LEA manifest interesting changes with respect to the model chromophore in solution. The most striking difference is the ~1522 cm^−1^ mode that is most intense in photoconverted LEA (Figure 10c) but is much weaker in various solvents (Figure 10e). The intensity difference is unlikely caused by the aforementioned vibronic coupling effect. The second-derivative analysis of the electronic absorption spectrum (Figure S10 in the Supplementary Materials) reveals a strongly coupled mode at ~1350 cm^−1^, corresponding to the 1368 cm^−1^ mode in the LEA protein that shows a large intensity due to the vibronic coupling effect (also the second strongest mode for the Kaede model chromophore in solution within the detection spectral window, Figure 10c). Therefore, the dramatic intensity change of the 1522 cm^−1^ mode likely results from the much increased polarizability derivative (∂α/∂Q, α is the electric polarizability and Q is the vibrational coordinate) which involves the styryl imidazole C=C/C=N stretching and I-ring C=N/C=O stretching motions (Table S5), induced by the chromophore–environment interactions, particularly at the I-ring extended sidechain site in the protein matrix [13,51,89].

### 2.4. Effects of the Chromophore Conformation

#### 2.4.1. GFP Model Chromophores: *cis* vs. *trans* Isomers

**Figure 11:**
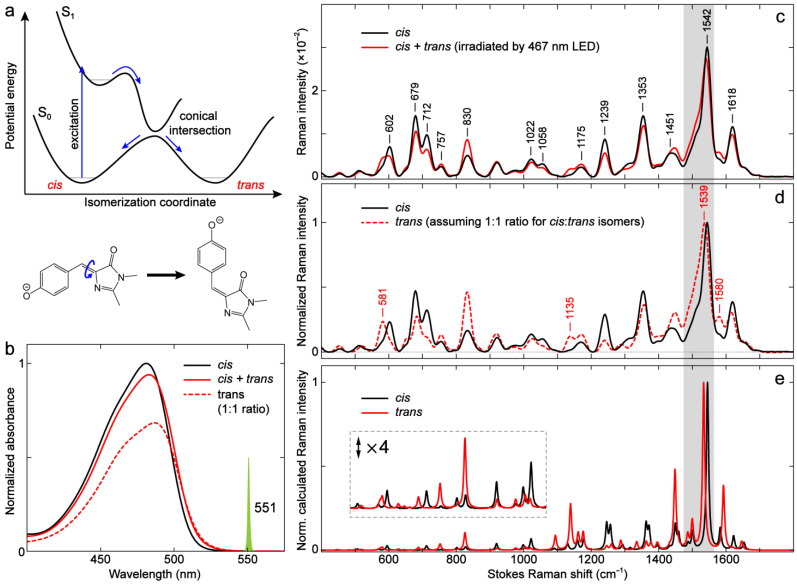
Characterization of the anionic *p*-HBDI of different conformations in DMSO. (**a**) General potential energy diagram along the exocyclic double-bond isomerization coordinate for the anionic *p*-HBDI. The *cis* and *trans* forms are denoted near the ground-state wells. (**b**) Steady-state electronic absorption spectra of the anionic *p*-HBDI in DMSO before (black solid) and after (red solid) 467 nm LED irradiation. The photostationary *cis* + *trans* mixture spectrum (red solid) was obtained by LED irradiation for 10 s; the spectrum is invariant after 10 s. The dashed spectrum (red) was obtained by subtracting 50% of the *cis* spectrum (black solid) from the mixture spectrum (red solid), followed by intensity doubling for a direct comparison with the *cis* spectrum. (**c**) Ground-state FSRS spectra of the anionic *p*-HBDI in DMSO before (black solid) and after (red solid) 467 nm LED irradiation. The *cis* + *trans* mixture (red solid) spectrum was obtained by constantly irradiating the sample during the measurement. The Raman pump (R_pu_) spectral profile at 551 nm is scaled and shown in (**b**) as a green spike. The FSRS spectra are collected on the Stokes side with respect to the R_pu_ center wavelength. The chromophore is deprotonated in DMSO by adding 0.05–0.1% (*v*/*v*) DBU. (**d**) Ground-state FSRS spectra of the *cis* (black solid) and *trans* (red dashed) anionic *p*-HBDI in DMSO. The *trans* spectrum is obtained by subtracting 50% of the *cis* spectrum from the mixture spectrum in (**c**), assuming that 50% *cis* and 50% *trans* chromophores are generated after photoisomerization. (**e**) Calculated Raman spectra of the *cis*- and *trans*-anionic *p*-HBDI in DMSO. The low-frequency region below ~1100 cm^−1^ is magnified four-fold to manifest the conformation-dependent Raman peak pattern change of the chromophore. The high-frequency marker band is highlighted by the gray rectangular shade across panels (**c**–**e**). See Section 3.4 for details of the quantum calculations.

The *cis*–*trans* photoisomerization is an important attribute of the FP chromophore and dictates the fluorescence efficiency for FPs in most cases. However, the pertinent vibrational characteristics of the *cis* and *trans* chromophores in either the protein matrix or solvents have rarely been compared and discussed. At thermal equilibrium, the anionic chromophores of most reported GFPs are in the *cis* conformation, while in very few cases, the *trans* conformation is stable over the *cis* conformation. For example, the positive [33,37] photoswitchable FP Padron0.9 adopts a *trans*-anionic chromophore that absorbs at 504 nm in its equilibrium off-state (i.e., nonfluorescent or having very low FQY) [100]. Another GFP with a thermally stable *trans*-anionic chromophore was reported by Kent et al., who found that the truncated GFP with the 11th β-strand removed forms a *trans*-anionic chromophore after refolding [101]. Unfortunately, these FPs are not readily available, and we therefore focus our investigation on the GFP model chromophore (i.e., *p*-HBDI) with extensive supporting literature (see examples above). Figure 11a illustrates the general potential energy diagram of the double-bond photoisomerization process for anionic *p*-HBDI [59,60,61,72,73]. Photoexcitation of the *cis* chromophore populates the excited state, which quickly undergoes isomerization through a peaked conical intersection (CI) [60,102] and relaxes to the original *cis* and/or *trans* conformation. Because the process is very efficient, anionic *p*-HBDI in solution has a very low FQY and is essentially nonfluorescent. The generation of the *trans* isomer upon excitation of the *cis* chromophore has been previously verified by ^1^H-NMR spectra [56,103,104].

Notably, most previous electronic absorption and ^1^H-NMR studies on the *cis–trans* photoisomerization of *p*-HBDI and its analogs have focused on the neutral form [22,103,104]. Besides confirming the photoisomerization process, these studies also revealed a significant solvent dependence for the *cis* isomer recovery rates. Since the *cis* chromophore is thermally more stable, while the light-induced *trans* isomer needs to overcome an activation energy barrier for interconversion, reflected by its long *trans*-to-*cis* recovery time in the ground-state (e.g., 3–5 min in D_2_O and ~48 h in methanol/isopropanol at room temperature) [56], the *trans* yield after photoisomerization is much higher in aprotic solvents than in protic solvents (i.e., the *trans* isomer becomes more stabilized in aprotic solvents). Yang et al. proposed that the yields for *cis* and *trans* isomers of neutral *p*-HBDI after going through a CI (Figure 11a) are evenly split (50/50%) by reasoning that the CI is located at a bridge C=C bond twisted angle of 90° [105], and they verified this assumption with ^1^H-NMR by showing that the yield of the *trans* isomer is ~50% in aprotic solvents (e.g., MeCN, tetrahydrofuran or THF, hexane) [103]. The ^1^H-NMR work by He et al. confirmed the photoisomerization of anionic *p*-HBDI, but since it was measured in water with an efficient thermal *trans*-to-*cis* (*E*→*Z*) isomerization, only ~2% of the *trans* isomer was detected [56]. We hereby chose to study anionic *p*-HBDI in the aprotic solvent, DMSO, to presumably generate an appreciable amount of *trans* isomer following photoisomerization at room temperature.

Upon 467 nm LED irradiation of the anionic *p*-HBDI in DMSO solvent, the photoisomerization indeed occurs and leads to a redshift in the electronic absorption band (Figure 11b) due to the generation of the *trans* isomer that results in a *cis* + *trans* mixture. This result suggests that the *trans* isomer absorbs slightly redder than the *cis* isomer (also see the *trans* spectrum in the red dashed line, Figure 11b, assuming a 1:1 ratio of *cis* and *trans* isomers). The neutral *p*-HBDI has also been reported to red-shift its electronic absorption peak in response to the excitation of the *cis* isomer [104]. To further examine the isomeric chromophore’s difference, we measured the ground-state FSRS spectra of a pure *cis* and *cis* + *trans* mixture with the same R_pu_ wavelength at 550 nm (Figure 11c). The mixture spectrum was obtained with a constant irradiation of 467 nm LED during the FSRS measurement (see Materials and Methods, Section 3.3). As shown in Figure 11c, the mixture spectrum differs from the *cis* spectrum across a broad frequency range but mainly in mode intensity (i.e., mode frequencies are largely conserved). In particular, the modes in the regions of 550–900 and 1100–1300 cm^−1^ exhibit relatively larger intensity changes than others. To retrieve the pure *trans* spectrum, one can typically use ^1^H-NMR spectra to determine the percentage of photogenerated *trans* isomer [56,103,104,106]. We herein aim to provide more of a qualitative understanding and reasonably assume, with prior ^1^H-NMR evidence for *p*-HBDI in aprotic solvents [103], that the *trans* isomer under constant LED irradiation is about 50% of the mixture in DMSO. The presumptive *trans* spectrum is thus obtained by subtracting half of the *cis* spectrum from the mixture (Figure 11d). In fact, we found from spectral data analysis that the *trans* spectrum would have negative Raman intensities if the percentage of *trans* isomer was higher than ~50%, which would contradict the positive line shape in the ground-state Stokes FSRS signal with an off- or preresonance R_pu_ wavelength [31,54,107]. This result implies that for the anionic *p*-HBDI in DMSO, the *trans* isomer can be generated as much as the *cis* isomer in the photostationary state mixture.

The *trans* spectrum (Figure 11d) displays several notable changes compared to the *cis* spectrum. The *trans* chromophore has considerably larger intensities than the *cis* chromophore for modes at ~581, 757, 830, 1135, 1175, 1451, and 1580 cm^−1^, if the spectra are normalized at the most intense mode at ~1540 cm^−1^. For a direct comparison, the *cis* chromophore modes at ~602, 679, 712, 1058, 1239, and 1353 cm^−1^ are more intense than those of the *trans* isomer. Interestingly, the dominant *cis* mode at 1542 cm^−1^ slightly red-shifts to 1539 cm^−1^ for the *trans* isomer (Figure 11d), which exhibits the aforementioned correlation between a red-shifted electronic absorption peak and a red-shifted quinoidal stretching mode frequency (see Figure 2a and Figure 3 for the –Me vs. –H substitutions and Figure 5a,b for the anionic *p*-HBDI *cis* isomer in water/MeCN/DMSO), thus corroborating the general sensitivity of this Raman marker band to the electron density distribution across the *p*-HBDI framework. We also note that the use of a redder Raman pump (551 nm in Figure 11c vs. 540 nm in Figure 5b) allows a cleaner spectral region from ~650 to 700 cm^−1^ for the chromophore modes to be studied, demonstrating the power of tunable FSRS technology in enhancing the solute signal while allowing a clear subtraction of the solvent/buffer/background signal [54,91]. These observed changes between the *cis*- and *trans*-anionic *p*-HBDI show excellent agreement with the calculated spectra across the entire detection window (Figure 11e). A recent theoretical work on the calculated Raman spectra of *cis*- and *trans*-anionic *p*-HBDI at an excitation wavelength of 514.5 nm also reported similar differences in mode intensity and frequency [108]. These correlated results provide strong evidence for an appreciable percentage of *trans* isomer in anionic *p*-HBDI being generated by photoisomerization in DMSO. No significant difference in the mode frequencies indicates that the *cis* and *trans* conformations of *p*-HBDI have similar electronic properties such as electron density distribution, though the observed mode-dependent nature still allows several Raman marker bands at ~581, 1135, 1539, and 1580 cm^−1^ for the *trans* isomer (labeled in Figure 11d).

#### 2.4.2. DsRed-Like RFP: *cis* vs. *trans* Isomers

**Figure 12:**
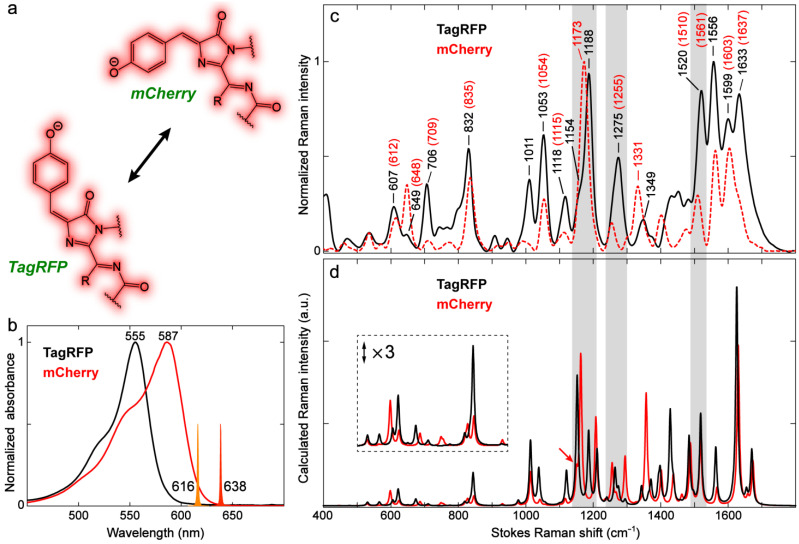
Characterization of DsRed-like RFPs with chromophores in *cis* and *trans* conformations. (**a**) Chromophore structures of mCherry and TagRFP. (**b**) Steady-state electronic absorption spectra of mCherry (red) and TagRFP (black) in aqueous buffers. The Raman pump (R_pu_) spectral profiles at 638 nm for mCherry and 616 nm for TagRFP are scaled and shown as color-coded spikes. (**c**) Ground-state FSRS spectra of mCherry (red dashed) and TagRFP (black solid) with prominent Raman modes labeled. The mCherry peak frequencies are in parentheses. The FSRS spectra were collected on the Stokes side with respect to the R_pu_ center wavelength. (**d**) Calculated Raman spectra of mCherry (red) and TagRFP (black) chromophores in water. See Materials and Methods for the calculation details. The low-frequency region from ~500 to 950 cm^−1^ is magnified three-fold to manifest the conformation-dependent Raman peak pattern change of the RFP chromophore. Some key mode changes are highlighted by gray rectangular shades across panels **c** and **d** for a clear comparison between the observed and calculated spectra.

Compared to GFPs, the presence of a *trans* chromophore in RFPs is not rare. RFPs with a *trans* chromophore or *cis* + *trans* mixed chromophores are most common in DsRed-type FPs, i.e., the chromophore has an extended conjugation by an acylimine moiety [12,50,109]. We chose a bright RFP, TagRFP, with the anionic chromophore exclusively in the *trans* conformation (Figure 12a) [50] to investigate the conformational effect on the vibrational properties with an extended sidechain as part of the chromophore conjugation network. TagRFP can also serve as a useful sample for comparison to mCherry with a *cis* chromophore (identical I-ring R_2_ sidechain) to shed essential light on the difference between the *cis* and *trans* RFP chromophores. The corresponding model chromophores were not studied because they are not synthesizable outside the protein matrix [95].

TagRFP absorbs at a bluer wavelength (555 nm) than mCherry (587 nm, Figure 12b), which can be largely attributed to their different local environments in the protein pockets. The environmental impact on the red chromophore can be seen from a recent work that reported a brighter mCherry mutant, mCherry-XL, created by mutating four residues near the RFP chromophore [110]. The mutations result in a three-fold enhancement of FQY for mCherry but blue-shifts it from 587 to 558 nm in electronic absorption, which is reminiscent of a recent report on an engineered brighter RFP with a noncanonical chromophore [95]. To discern the structural difference, the ground-state FSRS spectra under preresonance conditions were carefully compared. We first note that the relative intensities of high-frequency modes above 1400 cm^−1^ are generally higher in TagRFP than those in mCherry. The modes below 1400 cm^−1^ demonstrate mode-dependent intensity variations in the two RFPs (Figure 12c). The intensity difference can be caused by either the specific chromophore–environment interactions that intrinsically alter the polarizability derivative for different modes (see Section 2.3.2 above) or the chromophore conformation, which are intimately related inside a protein pocket due to the conformational change affecting both the chromophore itself as well as its immediate vicinity (i.e., local environment) [26,31].

Similarly, specific chromophore–environment interactions in FPs can also induce frequency shifts for the chromophore modes, which complicates the analysis of pure conformational effects. We therefore performed DFT calculations for the model chromophores to assist the analysis (see Figure S11 and Table S6 in the Supplementary Materials). Three high-frequency modes at 1556, 1599, and 1633 cm^−1^ in TagRFP are red-shifted from their counterparts in mCherry by 4–5 cm^−1^ (Figure 12c). These shifts are well predicted by the calculations, suggesting that the *trans* RFP chromophore has slightly lower frequencies than the *cis* chromophore for these double-bond stretching modes (Figure 12d). A similar frequency redshift in the quinoidal stretching mode (1556 cm^−1^ in TagRFP, which is a conserved vibrational normal mode across different model chromophores and FPs, see above) is also observed for the *trans*-anionic *p*-HBDI (Figure 11c,d). Interestingly as an opposite trend, the ~1520 and 1275 cm^−1^ modes in TagRFP represent a pronounced blueshift from the corresponding ~1510 and 1255 cm^−1^ in mCherry, respectively (Figure 12c). Whether the large blueshifts solely result from the conformational difference is debatable as our calculations show that these two normal-mode motions are slightly or moderately changed from the *cis* to *trans* isomer (see Table S3 for mCherry vs. Table S6 for TagRFP).

Another conspicuous difference between TagRFP and mCherry is the intense mode at ~1170–1190 cm^−1^. A single strong band at 1173 cm^−1^ is observed in mCherry, whereas dual peaks at 1154 and 1188 cm^−1^ are resolvable in TagRFP (Figure 12c). A similar phenomenon has also been observed for eqFP611 with a *trans* chromophore and DsRed with a *cis* chromophore [111]. The frequency difference is consistent with the calculated spectra (see the shaded regions in Figure 12d). The dual bands of close frequencies can be assigned to the P-ring and methine bridge H-rock, as well as the I-ring C–N stretch (Table S6 in the Supplementary Materials), while the two modes only differ in the relative phase between the P-ring and methine bridge H-rocking motions (see different directions of pertinent motions in Figure S12, Supplementary Materials). In fact, our current level of calculations also predict the presence of these two modes with very close frequencies for mCherry, but the lower-frequency one has a much weaker intensity (see the red arrow in Figure 12d). This point may explain the observation of a single Raman band in mCherry considering the relatively broad bandwidth in FSRS (vs. the narrower natural linewidth using spontaneous Raman, for example, but the signal strength with the fluorescence background is problematic therein) [54,91].

However, we note that the calculated Raman spectra of the model chromophores are overall in poor agreement with the experimental spectra of the two RFPs (Figure 12c,d). The simplified treatment of the model chromophore in water cannot sufficiently account for the complex chromophore–environment interactions inside the protein matrix, especially for a conjugated and extended sidechain at the I-ring end that essentially increases the interplay between the chromophore (larger in size) and its surroundings (with both electrostatic and steric effects) [5,15,19,26,88,93,95,112]. In other words, although TagRFP and mCherry demonstrate substantial difference in Raman modes, the lack of a complete understanding of the environmental effects makes it inconclusive to attribute the vibrational mode difference solely to the chromophore conformation. For example, the C=O bond within the acylimine moiety could be out of the chromophore plane, which reduces conjugation and the expected bathochromic shift [109,113], while forming new specific H-bonds with the protein residues in proximity. High-level quantum calculations and systematic tuning of the RFP local environment are expected to provide further insights into the conformational effects for RFP chromophores.

## 3. Materials and Methods

### 3.1. Protein Expression and Model Chromophore Preparation

#### 3.1.1. EGFP, mKO2, and TagRFP

The *E. coli* cells of the XL-1 Blue strain (Invitrogen, part of Thermo Fisher Scientific, Waltham, MA, USA) were used for FP expression. Lysogeny broth (LB) cell growth medium (200 mL in 2 L conical flasks) was completed with ampicillin (200 µg/mL, 500 µg/mL for mKO2) and inoculated with a single colony from a Petri dish 24 h after cell transformation with Y514 (carrying EGFP and TagRFP, pT5-6xHis-TEV-<LacZ_cassette_v2>-tL3S2P21|ori_pBR322|AmpR_cassette by Cloning Facility, Moscow, Russia) or pRSET-B (mKO2, Addgene, Watertown, MA, USA) expression vectors with an N-terminal cleavable 6xHis tag. Cell biomasses underwent 24 h growth at 37 °C in an Excella E25 (New Brunswick Scientific, part of Eppendorf, Edison, NJ, USA) thermostated shaker at 220 rpm, followed by 24 h growth at room temperature and 140 rpm for protein maturation.

Cell pellets were resuspended in a phosphate-buffered saline (PBS, pH 7.4, GIBCO, Thermo Fisher Scientific, Waltham, MA, USA) with 1 mM phenylmethylsulfonyl fluoride (PMSF, Sevicebio, Wuhan, China) after centrifugation via an Eppendorf Centrifuge 5415R (16.1 krcf for 25 min at 4 °C, precooled) and kept in an ice-water bath. Subsequently, sonication was performed using a Sonic Dismembrator (Fisher Scientific, part of Thermo Fisher Scientific, Waltham, MA, USA) for 15 min total treatment time (35 cycles of 6 s sonication at 30% input power amplitude and 20 s in off-cycle, Sonics Vibra-Cell, tapered microtip ¼”, Part No. 630-0420). Proteins were then purified using TALON metal-affinity resin (Clontech Laboratories, Takara Bio USA, Inc., Palo Alto, CA, USA) loaded in a 70% EtOH-washed glass column (~25 mL total volume, 2 mL of suspension) and equilibrated with 10× volume of PBS. The equilibration mixture was then replaced with fresh precooled PBS. The protein-containing liquid fraction of cell lysate was added stepwise to observe protein immobilization on TALON resin. The immobilized protein was then rinsed with 10× volume of PBS and washed with 10× volume of 10 mM imidazole in PBS. Further elution was performed with 250 mM imidazole in PBS.

The eluted protein concentration was estimated on the basis of each protein solution absorbance using a Cary 100 UV/Visible spectrophotometer (Agilent, Santa Clara, CA, USA) and known extinction coefficients from an FP-base database [114]. Each measurement was taken in PBS right after elution. Desalting was performed by ultrafiltration against a fresh PBS buffer using an Amicon Ultra, 10 KDa, 0.5 mL filter unit (Ultracel) in a way to reach a final imidazole concentration far below 1 mM. The volume of resulting solution was adjusted to obtain 1 mL of solution with a concentration range of 7–10 mg/mL. Each sample never reached concentrations above 12–15 mg/mL to avoid aggregation. The EGFP, mKO2, and TagRFP samples had final concentrations of ~3.9, 4.1, and 3.5 mg/mL, respectively, for the spectral data collection in this work.

The sodium dodecyl-sulfate polyacrylamide gel electrophoresis (SDS-PAGE) analysis was performed on the FPs to achieve the high-resolution separation of protein mixtures and confirm the targeted FP identity. Each protein sample of 0.25 µL volume was fully denatured by 10 min incubation at 95 °C in a buffer containing 8 M urea, 2% 1,4-dithiothreitol (DTT), 4% SDS, 50 mM tris(hydroxymethyl)aminomethane (Tris)-Cl, 2% glycerol, and 0.01% bromophenol blue. A 15% PAAG gel electrophoresis (Bio-Rad Mini-PROTEAN Tetra system chamber powered by Elf-4 power source from DNA-Technology, Moscow, Russia) was run at 80 V for 1 h and at 100 V for 1 h, until bromophenol blue bands left the gel completely. Gel fixation was followed with Coomassie Brilliant blue G-250 staining applied for 30 min and mQ gel washing for 24 h.

#### 3.1.2. mPapaya1, mOrange2, LEA, and KFP1

The pBAD vectors containing mPapaya and mOrange2 with N-terminal 6xHis affinity tags were purchased from Addgene, Watertown, MA, USA (Plasmids #54838 and #54531) and transformed into chemically-competent *Escherichia coli* (*E. coli*) Top10 cells, with transformants selected by plating on Luria–Bertani (LB) broth + 1.5% *w*/*v* agar + 50 µg/mL carbenicillin. Single colonies were used to inoculate 5 mL LB + 50 µg/mL carbenicillin liquid cultures that were shaken overnight at 37 °C and 250 rpm. The 5 mL overnight cultures were transferred to 1 L of LB + 50 µg/mL carbenicillin, and growth continued at 37 °C and 250 rpm until the optical density at 600 nm (OD_600nm_) reached ~0.5. The cultures were then transferred to a shaker at 30 °C (250 rpm) and growth continued until the O.D.600 reached ~0.6. L-arabinose was added to a final concentration of 0.2% *w*/*v*, and growth continued for ~12 h at 30 °C (180 rpm). Cells were pelleted by centrifugation (10,628× *g*) for 10 min. The cell pellets were thoroughly suspended in 50 mM Tris-HCl pH 8 + 10 mM imidazole pH 8 (4 mL/gram of cell pellet) and then frozen at −80 °C. Frozen pellet suspensions were thawed on ice, and 100 mM PMSF in isopropyl alcohol was added to a final concentration of 1 mM prior to cell lysis by sonication. Lysed cells were pelleted by centrifugation at 48,888× *g* and 4 °C for 45 min. To the supernatant, 5 M NaCl and 1 M DTT were added to a final concentration of 150 mM and 1 mM, respectively. Protein was purified over a 5 mL HisTrap Ni-NTA agarose column (Cytiva, Global Life Sciences Solutions USA LLC, Marlborough, MA, USA), washed with Ni-NTA buffer (50 mM Tris-HCl pH 8, 150 mM NaCl, 1 mM DTT) + 100 mM imidazole pH 8 to remove the weakly-bound contaminants, followed by elution in a Ni-NTA buffer + 500 mM imidazole pH 8. Protein eluted in the 500 mM imidazole fraction was dialyzed into 25 mM Tris-HCl pH 8, 150 mM NaCl, and 1 mM EDTA, centrifugally concentrated, and dialyzed again into 50 mM of 4-(2-hydroxylethyl)piperazine-1-ethanesulfonic acid (HEPES) pH 7.9, 150 mM NaCl, and 0.1 mM EDTA.

LEA was expressed and purified as previously described [10] prior to dialysis into 50 mM HEPES pH 7.9, 150 mM NaCl, and 0.1 mM EDTA. A gBlock containing KFP1, the A143G variant of asFP595 [97], sequence back-translated and codon optimized for expression in *E. coli*, was purchased from Integrated DNA Technologies (Coralville, IA, USA) and cloned between the NdeI and XhoI restriction sites of pET29a(+) using a Gibson assembly. Transformation, protein expression and purification were conducted as with mPapaya and mOrange2 save that BL21*(DE3) chemically competent cells were used rather than Top10, carbenicillin was replaced with kanamycin, and induction was started with the addition of 1 mM isopropyl β-D-thiogalactoside (IPTG) rather than 0.2% *w*/*v* L-arabinose. Aliquots of mPapaya at 7.1 mg/mL (231 μM, see Figure 1b for mPapaya1 as a monomeric version of *Zoanthus* sp. YFP [44] that has a zFP538-like chromophore being studied in this work), mOrange2 at 16.8 mg/mL (547 μM, see Figure 1b for mOrange2 that has an mKO-like chromophore), LEA at 12.9 mg/mL (488 μM, see Figure 1b for the red form of LEA that has a Kaede-like chromophore), and KFP1 at 36.3 mg/mL (1.34 mM, see Figure 1b for KFP1 that has an asFP595-like chromophore) were stored at –80 °C prior to thawing for spectral data collection at room temperature (22 °C).

#### 3.1.3. Dronpa2 and mCherry

The expression and purification were performed according to the literature protocols for Dronpa2 [88] and mCherry [110,115], and the protein identities were confirmed by visible spectral properties [88,110,114]. Proteins were exchanged into a pH 8.0 buffer containing 50 mM Tris-HCl and 250 mM NaCl and prepared to a concentration of 6.2 mg/mL (Dronpa2) or 8.8 mg/mL (mCherry) for experimental use, which supported the high signal-to-noise ratios for these two proteins in ground-state FSRS characterization (see Figure 6 and Figure 8).

#### 3.1.4. mTFP0.7

The mTFP0.7 protein sample was prepared and purified according to the literature [42,116] following the engineering efforts to create a monomeric teal fluorescent protein (TFP) from the naturally occurring tetrameric coral reef *Clavularia* cyan fluorescent protein (CFP) and improve the versatility of CFPs in general. The pH 7.9 aqueous buffer solution consisted of 50 mM HEPES and 300 mM NaCl salt for protein spectral characterization in vitro.

#### 3.1.5. FP Model Chromophore Preparation

All the model compounds were previously obtained in our laboratory. The synthetic procedures, yields, and supporting spectral data including NMR can be found in our previous reports for all the *p*-HBI derivatives (see GFP model chromophores in Section 2.1) [117], KFP1/asFP595 model chromophore (see Section 2.3.1) [96], and the Kaede model chromophore (see Section 2.3.2) [118,119].

### 3.2. Steady-State Electronic Spectroscopy

The steady-state electronic absorption spectra of all the FPs and model chromophores in solution were collected by a Thermo Scientific Evolution 201 UV/Visible spectrophotometer at room temperature (22 °C). The solution sample was filled in a 1 mm pathlength quartz cuvette (1-Q-1, Starna Cells, Inc., Atascadero, CA, USA). The sample concentration was kept at an optical density (OD) below 3 per mm (typically above 1 per mm, see below) to avoid spectral signal saturation.

### 3.3. Femtosecond Stimulated Raman Spectroscopy (FSRS)

The ground-state FSRS spectra were collected using a home-built optical setup in a temperature- and humidity-controlled room. It consists of a picosecond (ps) narrowband Raman pump with broad wavelength tunability across the visible spectral range and a femtosecond (fs) broadband Raman probe from supercontinuum white light generation [120]. In brief, the Raman pump is generated by a two-stage ps noncollinear optical parametric amplifier (NOPA) with a tunable ps seed and a ps 400 nm pump. The Raman probe is generated by focusing a small portion of the ~800 nm fundamental pulse (FDP) onto a 2 mm pathlength quartz cuvette filled with deionized water, followed by temporal compression using a chirped mirror pair (DCM-9, 450–950 nm, Laser Quantum, Inc., part of Novanta Photonics, Stockport, UK). The FDP with ~35 fs duration and 1 kHz repetition rate is provided by a mode-locked Ti:sapphire oscillator seeding a laser regenerative amplifier (Legend Elite-USP-1K-HE, Coherent, Inc., Santa Clara, CA, USA). A detailed description of our FSRS setup in the mixed time–frequency domain can be found in our previous publications [21,91,92,107,120,121]. In particular, the spectral resolution of FSRS depends on the pulse duration of the Raman pump (~2 ps) and dephasing time of the generated vibrational coherence (typically hundreds of fs to a few ps), the latter of which usually limits the observed spectral resolution to about 10–15 cm^−1^ in our setup. All the FSRS spectra were calibrated using the DMSO and perdeuterated DMSO (DMSO-*d*_6_) solvent mixtures with a volumetric ratio of 1:1 that covers a relative broad frequency range of ~400–2250 cm^−1^ for an accurate resultant Raman-shift frequency axis. The Raman pump powers used for all ground-state FSRS measurements were kept at ~3–4 mW (or μJ/pulse). The concentrations of most FP samples and model chromophore solutions were set above 1 per mm in absorbance (OD) for FSRS measurements to achieve high signal-to-noise ratios, and the UV/Visible spectra were collected before and after FSRS experiments to confirm sample integrity, which is robust due to spectral data collection in the electronic ground-state [54,91,107]. The FSRS spectrum of each sample was acquired by 1500 × 50 = 75,000 spectral averaging (i.e., 3000 shots per scan with the Raman pump on and off, then 50 sets of scans) in a custom-built LabVIEW suite (National Instruments Corp., Austin, TX, USA) and smoothed through a 9-point averaging step in Igor Pro (WaveMetrics, Inc., Portland, OR, USA). No other denoising steps were employed.

### 3.4. Computational Methods

The calculations of all the ground-state Raman spectra and bond lengths were performed with density functional theory (DFT) at the B3LYP level of theory with 6-311G+(d,p) basis sets [122]. The chromophore geometry was optimized for the electronic ground-state, while the implicit solvent integral equation formalism polarizable continuum model (IEFPCM) was used. For ground-state Raman calculations of all the FP chromophores and model chromophores in water, an explicit water molecule was added in the immediate vicinity of the phenolate group to account for specific H-bonding interactions (e.g., see Figure 4 and Table 2), while the bulk water was still treated by the IEFPCM method. Additional scenarios of an explicit water molecule at the I-ring C=O end as well as two explicit water molecules at both the P-ring and I-ring ends were only considered for *p*-HBDI (see Table 2 and Figure S1d–f). No explicit solvent molecules were added for the calculations of model chromophores in MeCN and DMSO.

## 4. Conclusions

In this work, we used wavelength-tunable femtosecond stimulated Raman spectroscopy (FSRS) to characterize the ground-state vibrational spectra (without an actinic pump) for the deprotonated (anionic) chromophores of a wide variety of fluorescent proteins (FPs) with emission colors spanning a broad wavelength range, as well as the synthetic model chromophores for some of these FPs. Through systematic comparisons, we discussed four typical factors that affect the vibrational properties of the FP chromophores across an expanded palette. A pronounced double-bond stretching mode at ~1530–1565 cm^−1^ that is conserved in most FPs and model chromophores despite their structural variations is found to be a useful indicator for revealing chromophore–environment interactions and structural effects on the electronic properties of FP chromophores. This marker band results from stretches of the bonds characteristic of the quinoidal resonance structure. Its frequency shift in response to variations in the chromophore structure and local environment can be generally interpreted by a shift between the benzenoid and quinoid resonance structures of the chromophore, reflecting the electron density redistribution from the phenolate to imidazolinone ring moieties or vice versa.

First, the I-ring sidechain of the GFP model chromophore, being –H or alkyl groups, causes substantial changes in the observed vibrational normal modes. Depending on the substitution site, the frequency shift of the quinoidal stretching mode reflects a shift between the two resonance structures and is found to be in line with the change in the imidazolinone C=O bond length despite the mode being involved with stretches of other quinoidal double bonds. Second, the environment (solvents or protein matrices) poses influences on the FP or model chromophores and shifts the resonance structure from one toward the other through H-bonding and dipole–dipole interactions. Likewise, we found that the frequency shift of the quinoidal stretching mode can be qualitatively explained by the imidazolinone C=O bond length change, particularly for GFPs (i.e., with green emission), which can be attributed to the varied H-bonding interactions between local residues and the chromophore at the phenolate –O^−^ as well as imidazolinone C=O ends. Third, different FP chromophore structures with extended-conjugation sidechains such as –C=N and –C=N–C=O yield largely similar vibrational modes with small frequency shifts. Compared to GFPs, the quinoidal stretching modes in these redder FPs exhibit a frequency blueshift. This trend is ascribed to the electron redistribution from the phenolate to imidazolinone rings due to the substantial electron-withdrawing ability of the extended moieties, as corroborated by the detailed comparison between the GFP model chromophore, *p*-HBDI, and the asFP595 model chromophore in water. We also investigated a green-to-red photoconvertible FP which has a distinct chromophore structure in the converted form vs. other RFPs. The unique chromophore structure results in drastic changes in the normal modes with respect to the GFP chromophore. Last, the chromophore conformation, i.e., *cis* and *trans*, was found to impact the vibrational properties depending on the chromophore structure. The *cis* and *trans* isomers of the anionic GFP model chromophore, *p*-HBDI, are only slightly different, mainly in mode intensity. The comparison between two RFPs, mCherry and TagRFP, with the *cis* and *trans* chromophores, respectively, shows considerable differences in both mode intensity and frequency across the broad detection spectral window. However, the complication from the environmental effect makes it inconclusive to attribute the observed differences solely to the chromophore conformations.

The fruitful and comprehensive insights gained by the tunable FSRS technique for FP chromophore properties in the equilibrium electronic ground-state greatly complement previous studies of the chromophore structures and chromophore–environment interactions obtained via X-ray crystallography and steady-state electronic spectroscopy. The sophisticated nature of vibrational spectroscopy, especially Raman spectroscopy, with bond precision for the chromophore structure and little interference from the protein backbone due to the very weak resonance enhancement (i.e., essentially “transparent” protein residues except for the three-residue chromophore with preresonance enhancement in this work), makes it a highly useful toolset to delineate FP chromophore structures and conformations [51,95,111,123]. Beyond the use in ground-state characterization, ultrafast vibrational spectroscopy such as excited-state FSRS with a preceding actinic pump is powerful in tracking the transient nonequilibrium structural and population dynamics of FP chromophores in real time, thus enabling the capture of vivid “molecular movies” underlying the photophysical and photochemical functionalities in myriad FPs. This line of inquiry has seen increasing efforts in recent years [15,24,25,26,124,125], and we expect our work herein to set useful benchmarks for future investigations on more engineered FPs and nanoprobes with improved properties such as redder emissions, enhanced brightness, higher stability or switchability, and greater photochromic tunability in physiologically relevant environments for a multitude of bioimaging advances.

## Figures and Tables

**Table 1:** Calculated bond lengths of *p*-HBI with –H and –Me sidechain substituents in water *.

R_1_	R_2_	Bond Length (Å)						
		CO, 1	CC, 2	CC, 3	CC, 4	CC, 5	CO, 6	CN, 7
Me	Me	1.2823	1.3761	1.3759	1.4272	1.3711	1.2351	1.3023
Me	H	1.2800	1.3747	1.3744	1.4234	1.3738	1.2363	1.2984
H	Me	1.2818	1.3757	1.3755	1.4263	1.3715	**1.2340**	1.3008

* The calculations were performed with density functional theory (DFT) at the B3LYP level of theory and 6-311G+(d,p) basis sets. The chromophore geometry was optimized in the electronic ground state, while water was used as the implicit solvent using the integral equation formalism polarizable continuum model (IEFPCM). An explicit water molecule was added near the phenolate end to account for specific H-bonding interactions. The I-ring C=O bond length in a more benzenoid structure is bolded. The bond numbering scheme can be seen in the right panel of Figure 4.

**Table 2:** Calculated bond lengths of *p*-HBDI in different solvents *.

	Bond Length (Å)						
	CO, 1	CC, 2	CC, 3	CC, 4	CC, 5	CO, 6	CN, 7
water (implicit)	1.2709	1.3741	1.3735	1.4229	1.3747	1.2372	1.3026
water (1 explicit, –O^−^)	1.2823	1.3761	1.3759	1.4272	1.3711	1.2351	1.3023
water (1 explicit, C=O)	1.2688	1.3725	1.3720	1.4191	1.3783	1.2462	1.3019
water (2 explicit, –O^−^ and C=O)	1.2802	1.3747	1.3744	1.4237	1.3745	**1.2438**	1.3014
MeCN (implicit)	1.2703	1.3739	1.3733	1.4223	1.3750	1.2372	1.3024
DMSO (implicit)	1.2706	1.3740	1.3734	1.4226	1.3749	1.2372	1.3025

* The calculations were performed with density functional theory (DFT) at the B3LYP level of theory and 6-311G+(d,p) basis sets. The chromophore geometry was optimized in the ground electronic state, and the IEFPCM method was used to account for the implicit solvation effects. One or two explicit water molecules were added to the chromophore phenolate and/or I-ring carbonyl ends to account for specific H-bonding interactions in the immediate vicinity of the chromophore (see the optimized geometries in Figure S1d–f). The I-ring C=O bond length in the presence of two explicit water molecules is bolded for discussion (see main text). Note that the bond length values in Table 1 (see above) were obtained with one explicit water molecule added to the chromophore phenolate end, thus matching the second row of this table.

**Table 3:** Ground-state FSRS mode frequencies of *p*-HBDI in water and inside different GFP-like proteins *.

*p*-HBDI (Water)	*p*-HBDI (DMSO)	EGFP	Dronpa2	mTFP0.7	LEA (Green Form)
612	602	616	616	624	613
852	830	844	846	851	844
932	921	917	920	907	923
–	–	1079	1083	1071	1080
1164	1175	1163	1164	1161	1168
1238	1239	1256	1259	1248	1261
1302	1295	1324	1329	1318	1329
1363	1353	1362	1367	1365	**1365**
1496	1487	1492	1493	1492	1494
**1548**	**1542**	**1532**	**1532**	**1541**	**1531**
1578	1576	1570	1570	1574	1565
1623	1618	1614	1621	1622	1616

* The mode frequencies are in the cm^−1^ unit, and only prominent modes that appear in all four GFPs are shown for comparison. The R_pu_ wavelengths used in the ground-state Stokes FSRS measurement are 509 nm (anionic *p*-HBDI in water and mTFP0.7) and 540 nm (EGFP, Dronpa2, and LEA). The first two columns of spectral data in this table match those presented in Figure 3a (Table S1) and Figure 5b (Table S2) for the model GFP chromophore *p*-HBDI in solution. The most prominent Raman mode (except for being one of the two strongest peaks in LEA) is bolded for direct comparison across the samples.

## Data Availability

All data needed to evaluate the conclusions in the paper are present in the paper and the Supplementary Materials.

## Supplementary Materials

The supporting information can be downloaded at https://www.mdpi.com/article/10.3390/ijms241511991/s1.
