# Supplementary material for: Structural Characterization of Fluorescent Proteins Using Tunable Femtosecond Stimulated Raman Spectroscopy
Source: Int J Mol Sci. 2023 Jul 26;24(15):11991. doi: 10.3390/ijms241511991 (PMC10418586; doi:10.3390/ijms241511991)
Supplement: Supplementary file 1 [file ijms-24-11991-s001.zip › ijms-2499955-supplementary.pdf]

Supplementary Materials (SM) for:

Structural Characterization of Fluorescent Proteins Using Tunable Femtosecond Stimulated Raman Spectroscopy

Cheng Chen [1], J. Nathan Henderson [2], Dmitry A. Ruchkin [3], Jacob M. Kirsh [4], Mikhail S. Baranov [3,5], Alexey M. Bogdanov [3], Jeremy H. Mills [2,6], Steven G. Boxer [4], and Chong Fang [1,*]

[1] Department of Chemistry, Oregon State University, 153 Gilbert Hall, Corvallis, Oregon 97331, USA

[2] Center for Molecular Design and Biomimetics, The Biodesign Institute, Arizona State University, Tempe, Arizona 85287, USA

[3] Shemyakin-Ovchinnikov Institute of Bioorganic Chemistry, Russian Academy of Sciences, Ulitsa Miklukho-Maklaya, 16/10, 117997 Moscow, Russian Federation

[4] Department of Chemistry, Stanford University, Stanford, California 94305, USA

[5] Laboratory of Medicinal Substances Chemistry, Institute of Translational Medicine, Pirogov Russian National Research Medical University, Ostrovitianov 1, Moscow 117997, Russian Federation

[6] School of Molecular Sciences, Arizona State University, Tempe, Arizona 85287, USA

*To whom correspondence should be addressed. E-mail: Chong.Fang@oregonstate.edu.

Table of Contents

1. Supplementary Figures S3

Figure S1. Comparison between experimental and calculated Raman spectra of the deprotonated (anionic) *p*-HBI with –H and –Me substituents in water S3

Figure S2. Comparison between the experimental and calculated Raman spectra of the anionic *p*-HBDI in (a) MeCN and (b) DMSO S4

Figure S3. Second-derivative analysis of the electronic absorption spectra for the anionic *p*-HBDI in different solvents S5

Figure S4. (a) Second-derivative analysis of the electronic absorption spectra for different GFPs and (b-c) ground-state FSRS data of EGFP with tunable Raman pump (R_{pu}) wavelengths.......... S6

Figure S5. Electronic absorption spectra of the matured and unmatured mOrange2 chromophores S8

Figure S6. Comparison between the experimental and calculated Raman spectra of Y/O/RFPs . S9

Figure S7. Comparison between the experimental and calculated Raman spectra of *cis* anionic KFP1 model chromophore in water S10

Figure S8. Electronic absorption, excitation, and emission spectra of the photoconverted LEA protein S11

Figure S9. Comparison between the experimental and calculated Raman spectra of the anionic Kaede chromophore in (a) water, (b) MeCN, and (c) DMSO S12

Figure S10. Second-derivative analysis of the electronic absorption spectra for the anionic Kaede chromophore in different solvents and the photoconverted LEA protein S13

Figure S11. Comparison between the experimental and calculated Raman spectra of TagRFP. S14

Figure S12. Motions of the H-rocking modes at ~1150–1200 cm^{-1} in TagRFP S15

2. Supplementary Tables.. S16

Table S1. Mode assignment for the anionic *p*-HBI with –H and –Me substituents in water. S16

Table S2. Mode assignment for the anionic *p*-HBDI in two organic solvents S18

Table S3. Mode assignment for the deprotonated chromophores of mPapaya1, mKO2, mOrange2, and mCherry S19

Table S4. Mode assignment for the deprotonated *trans* chromophore of KFP1 .... S21

Table S5. Mode assignment for the deprotonated Kaede model chromophore and the photoconverted LEA chromophore S22

Table S6. Mode assignment for the deprotonated *trans* chromophore of TagRFP S24

1. Supplementary Figures

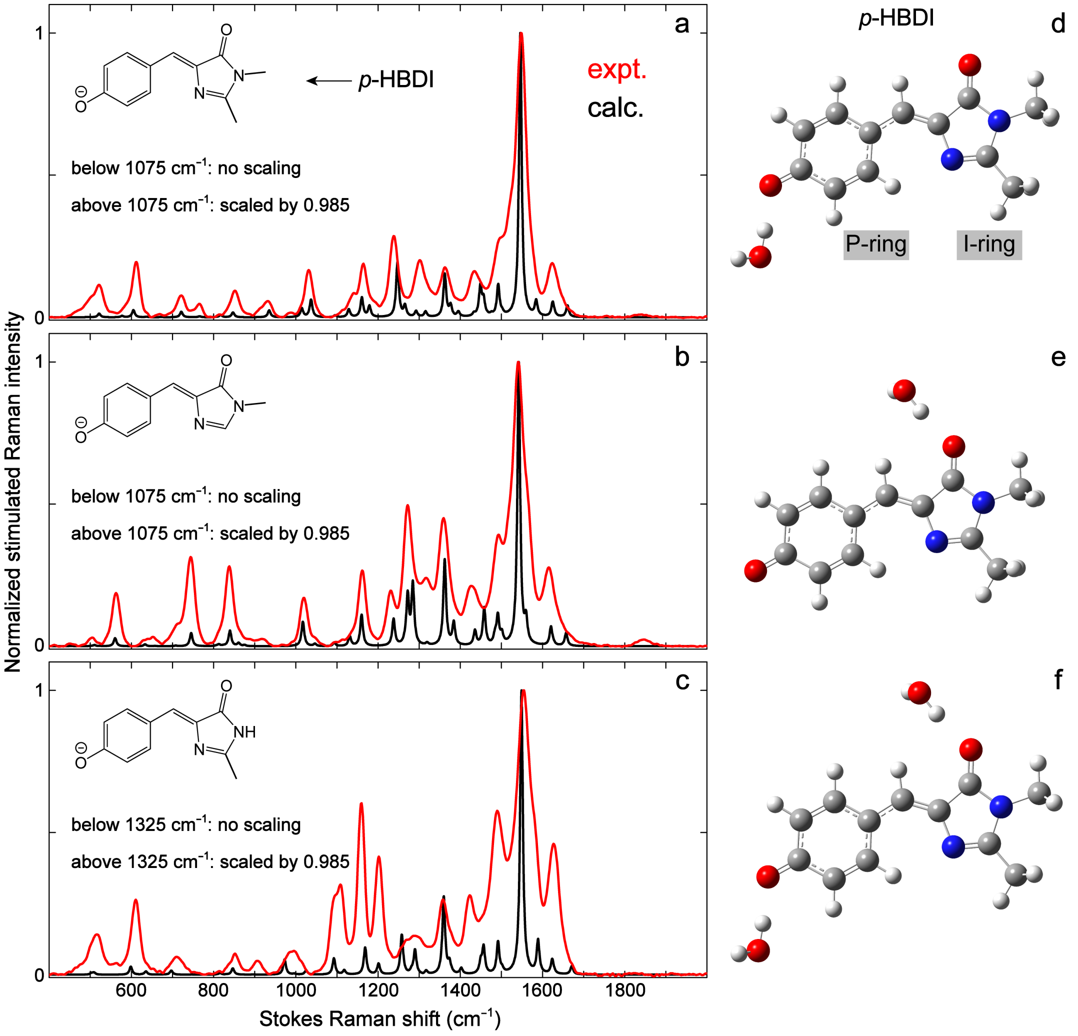

Figure S1. Comparison between experimental and calculated Raman spectra of the deprotonated (anionic) *p*-HBI with –H and –Me substituents in water. See Materials and Methods in main text for details of the experimental conditions and calculation methods. The frequency scaling factors and specific chromophore structures are shown in the insets of panels (**a**) to (**c**). The peak width (full width at half maximum or fwhm) of the calculated spectra (black) is set at 8 cm^{-1} for a visual comparison with the experimentally observed spectra (red) of these chromophores (see Table S1). The calculations performed with one or two explicit water molecules added at the P-ring and/or I-ring ends of *p*-HBDI chromophore lead to the optimized structures displayed in panels (**d**) to (**f**).

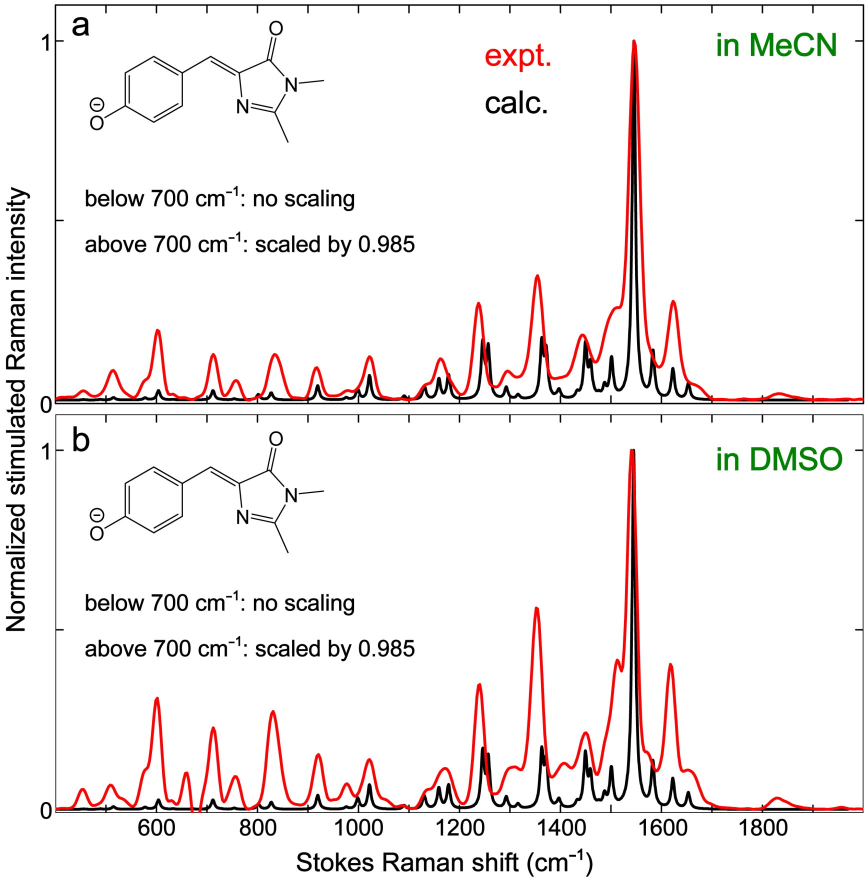

Figure S2. Comparison between the experimental and calculated Raman spectra of the anionic *p*-HBDI in (**a**) MeCN and (**b**) DMSO. See Materials and Methods in main text for details of the experimental conditions and quantum calculation methods. The frequency scaling factors and chromophore structures are shown in the insets. The peak width (fwhm) of the calculated spectra (black) is set at 8 cm^{-1} for a visual comparison with the experimentally observed spectra (red) of the chromophores in two solvents (see Table S2).

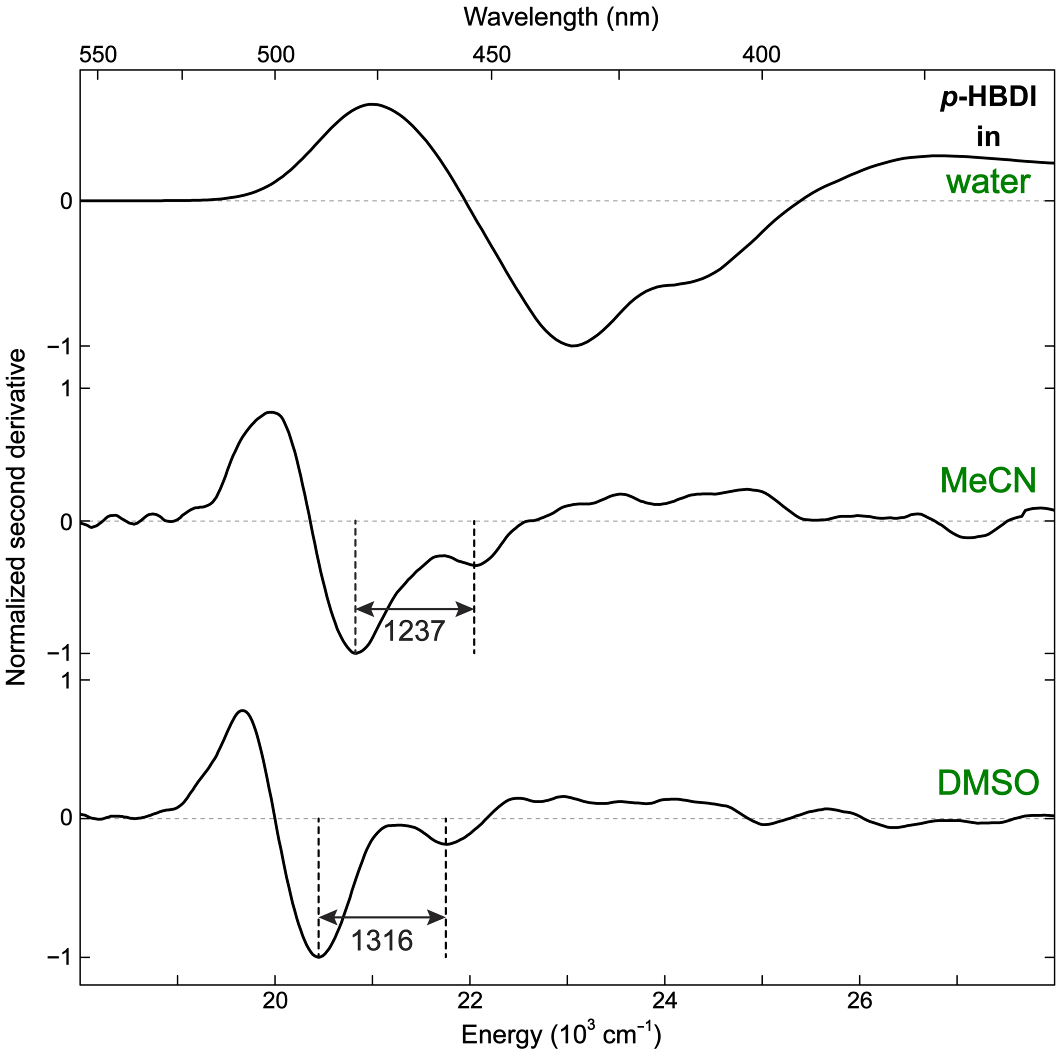

Figure S3. Second-derivative analysis of the electronic absorption spectra for the anionic *p*-HBDI in different solvents. The vibronically coupled mode frequency is estimated by the difference of the 0–0 and 0–1 transition peak energies (denoted by two vertical dashed lines) which are clearly resolvable in MeCN and DMSO. The energy and wavelength axes are shown below and above the spectral traces, respectively. See Figure 5a in main text for the associated absorption spectra.

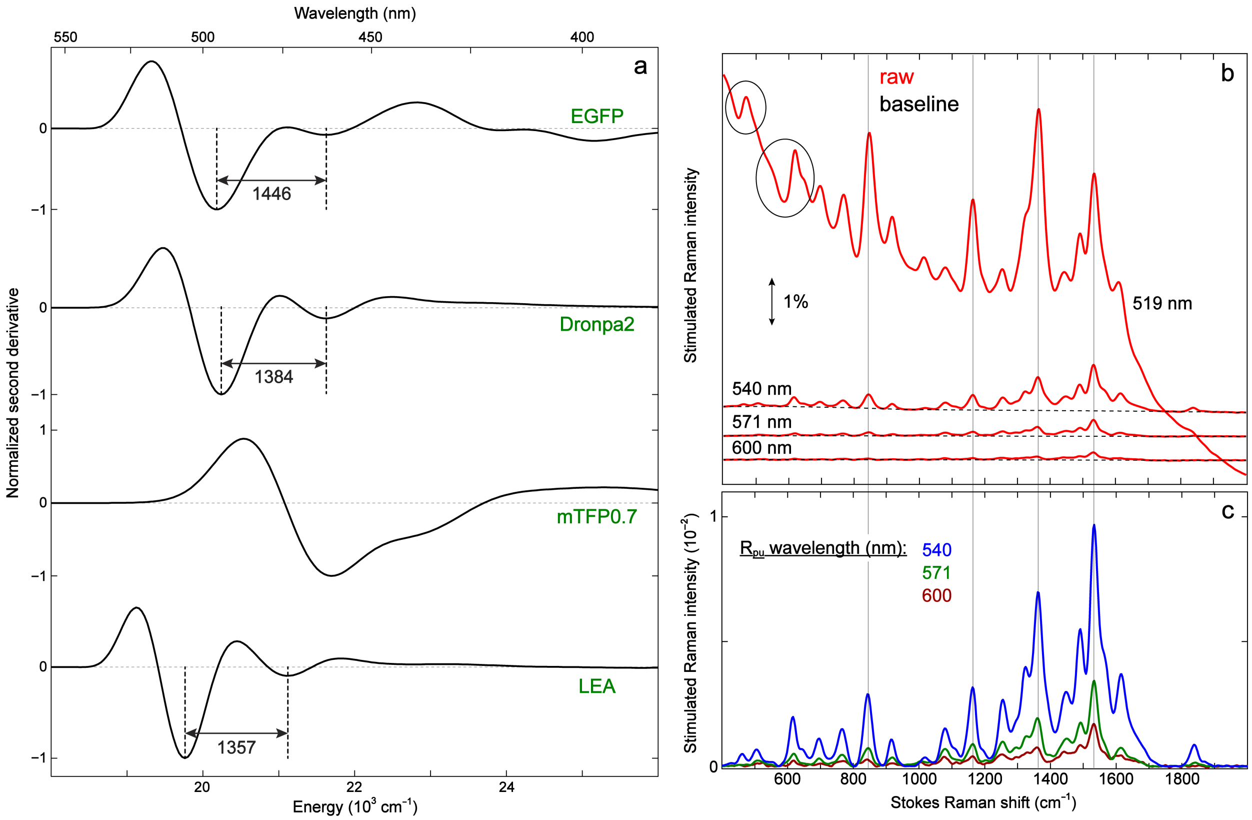

Figure S4. (**a**) Second-derivative analysis of the electronic absorption spectra for different GFPs and (**b-c**) ground-state FSRS data of EGFP with tunable Raman pump (R_{pu}) wavelengths. In panel (**a**), the vibronically coupled mode frequency is estimated by the difference of the 0–0 and 0–1 transition peak energies (denoted by two vertical dashed lines) which are clearly resolvable for EGFP, Dronpa2, and LEA (green form). The energy and wavelength axes are shown below and above the spectral traces, respectively. See Figure 6a in main text for the associated electronic absorption spectra. In panel (**b**), raw FSRS spectra (red solid lines) and baselines (black dashed lines) of EGFP in pH 7.4 buffer under different resonance conditions are shown. The on-resonance condition (see Figure 6a top panel for the absorption spectrum of EGFP) with a 519 nm R_{pu}, still redder than the 490 nm absorption peak, results in a worsened baseline and dispersive line shapes (circled, below 700 cm^{-1} for example). For comparison, the pre- to off-resonance conditions (R_{pu} wavelengths from 540, 571, to 600 nm) yield basically flat baselines. Panel (**c**) displays the

overlaid baseline-subtracted FSRS spectra of EGFP at pH 7.4 with R_{pu} wavelengths at 540 (blue), 571 (green), and 600 nm (maroon). To account for experimental variations (e.g., laser pulse intensity, beam spatial overlap) at different R_{pu} wavelengths, the spectra are scaled accordingly by normalizing (540 nm case as the reference) the peak intensity of the standard solvent for calibration, since the solvent Raman peaks can be considered being collected under off-resonance conditions in all cases. Gray thin lines mark a number of prominent Raman peaks with identical frequencies in the electronic ground state and increasing intensities as R_{pu} is tuned from off-resonance (600, 571 nm), pre-resonance (540 nm), to largely on-resonance (519 nm) conditions.

Notably, our current work has focused on a systematic analysis of Raman peak locations and intensities across a wide array of samples to draw conclusions (see main text). The other peak properties besides position and intensity are not directly related to our aim focusing on structural insights of the FP chromophores. For instance, the widths, asymmetries or shoulders of Raman peaks could stem from an inhomogeneous chromophore population with slightly different conformations or local environments, which may also display various degrees of excitation-light dependence. The detailed perusal of these properties can thus motivate future experimental and theoretical efforts which will deepen our understanding of chromophores in complex environments.

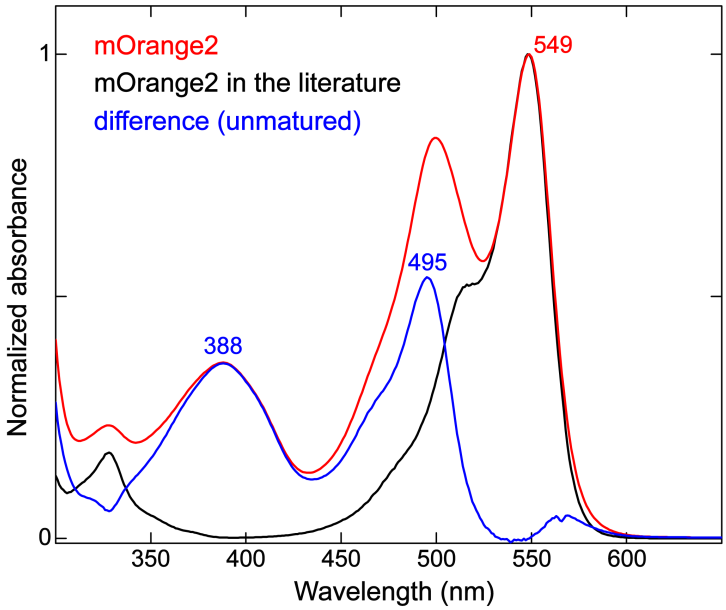

Figure S5. Electronic absorption spectra of the matured and unmatured mOrange2 chromophores. The spectrum of matured mOrange2 chromophore (black trace) was taken from the literature (also see fpbase.org/protein/morange2/). The unmatured chromophore (blue trace) shows two bands at ~388 and 495 nm, falling into the common wavelength regions of the protonated and deprotonated GFP chromophores, respectively.

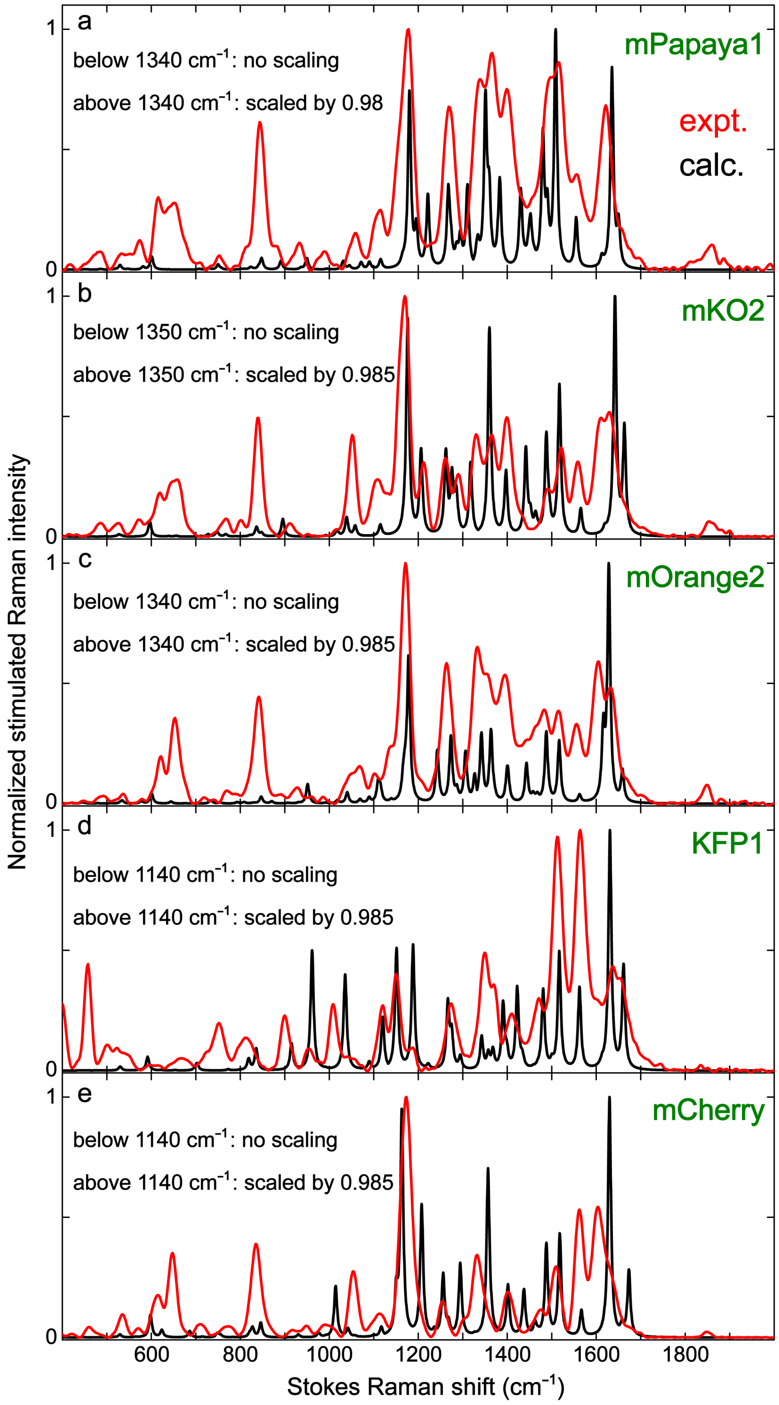

Figure S6. Comparison between the experimental and calculated Raman spectra of Y/O/RFPs. See Materials and Methods in main text for the experimental conditions and calculation methods. The frequency scaling factors are shown in (**a**)–(**e**) insets. The peak width of the calculated spectra (black) is set at 8 cm^{-1} for a visual comparison with the experimentally observed spectra (red).

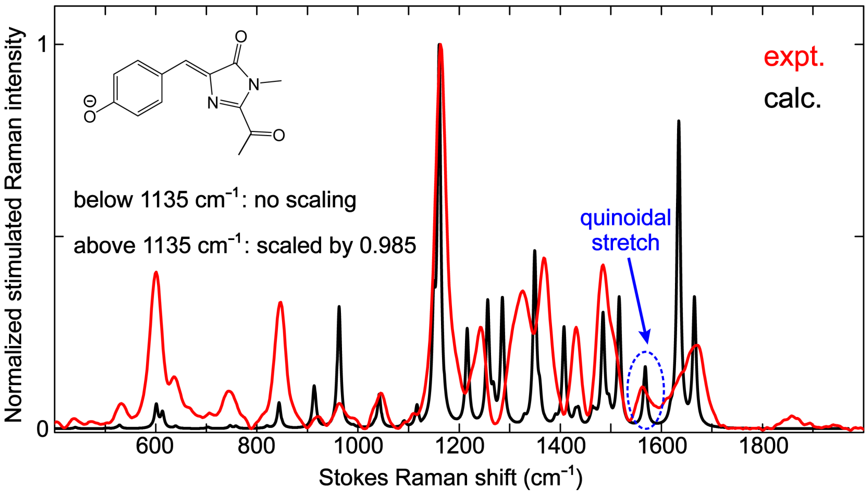

Figure S7. Comparison between the experimental and calculated Raman spectra of *cis* anionic KFP1 model chromophore in water. See Materials and Methods in main text for details of the experimental conditions and calculation methods. The chromophore chemical structure and frequency scaling factor are shown in the inset. The peak width of the calculated spectrum (black) is set at 8 cm^{-1} for a visual comparison with the experimentally observed spectrum (red). A key Raman marker band is circled by a dashed ellipse (see main text for discussions).

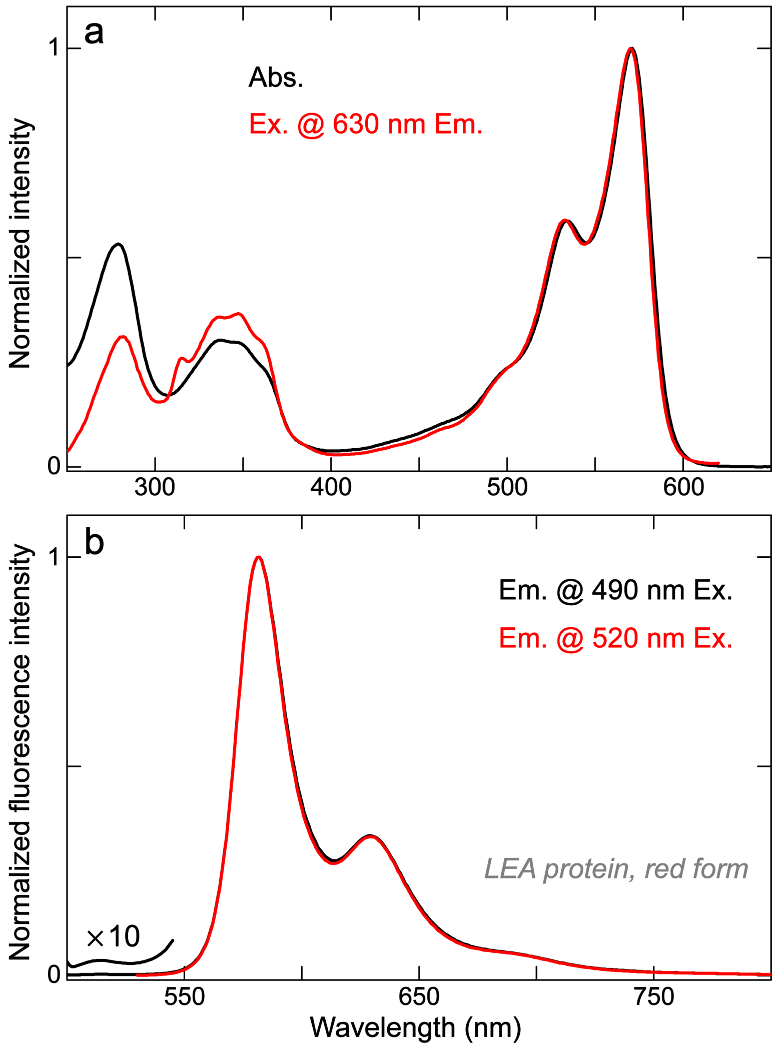

Figure S8. Electronic absorption, excitation, and emission spectra of the photoconverted LEA protein. (**a**) Comparison of the normalized absorption (Abs., black) and excitation (Ex., red) spectra of the photoconverted LEA in red form. The excitation spectrum was obtained with emission wavelength of 630 nm. (**b**) Comparison of the normalized emission (Em.) spectra of photoconverted LEA with excitation wavelengths of 490 nm (black) and 520 nm (red). The 500–550 nm region upon 490 nm excitation is characteristic of green emission from the unconverted LEA in green form, and is magnified tenfold to showcase the minimal green emission post the green-to-red photoconversion of LEA protein.

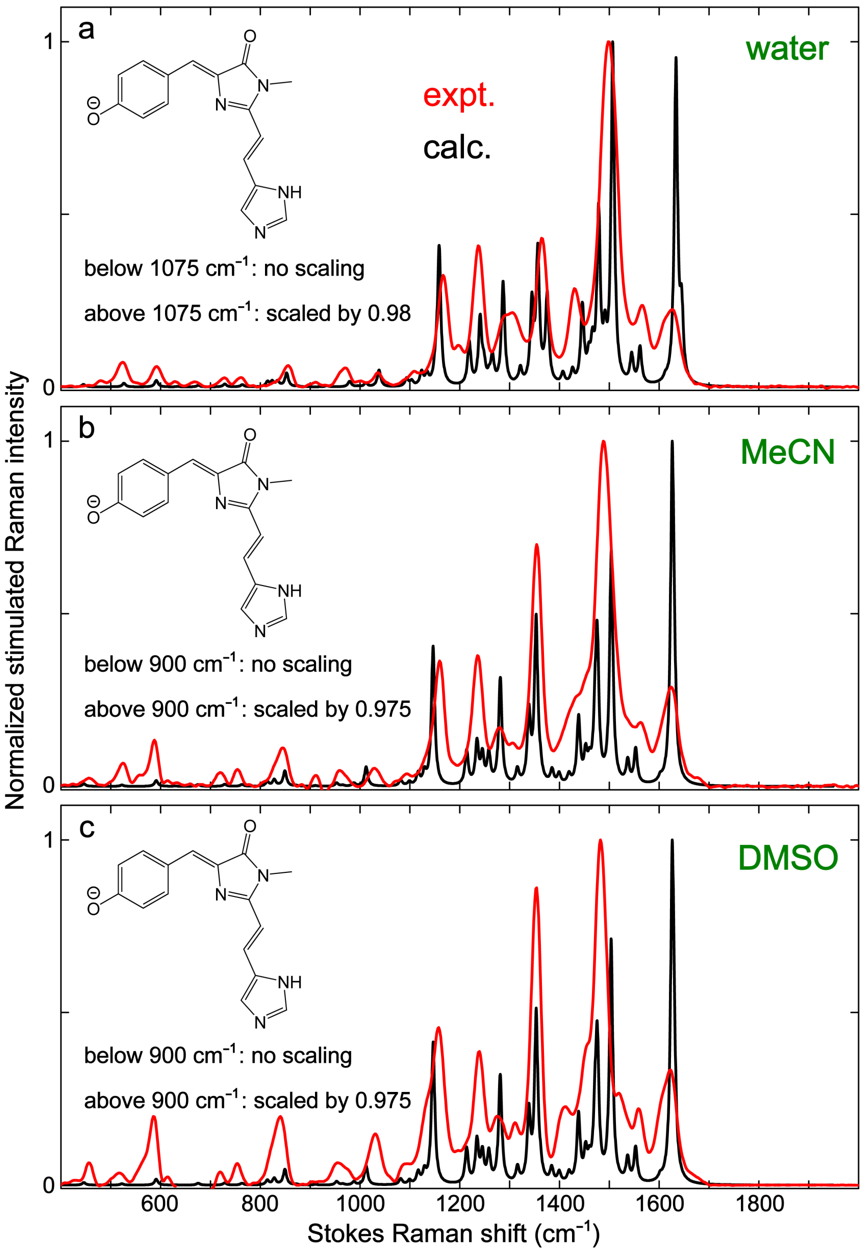

Figure S9. Comparison between the experimental and calculated Raman spectra of the anionic Kaede chromophore in (**a**) water, (**b**) MeCN, and (**c**) DMSO. See Materials and Methods in main text for details of the experimental conditions and calculation methods. The frequency scaling factors and chromophore chemical structures (the identical deprotonated Kaede model chromophore across three panels) are shown in the insets. The peak width of the calculated spectra (black) is set at 8 cm^{-1} for a visual comparison with the experimentally observed spectra (red).

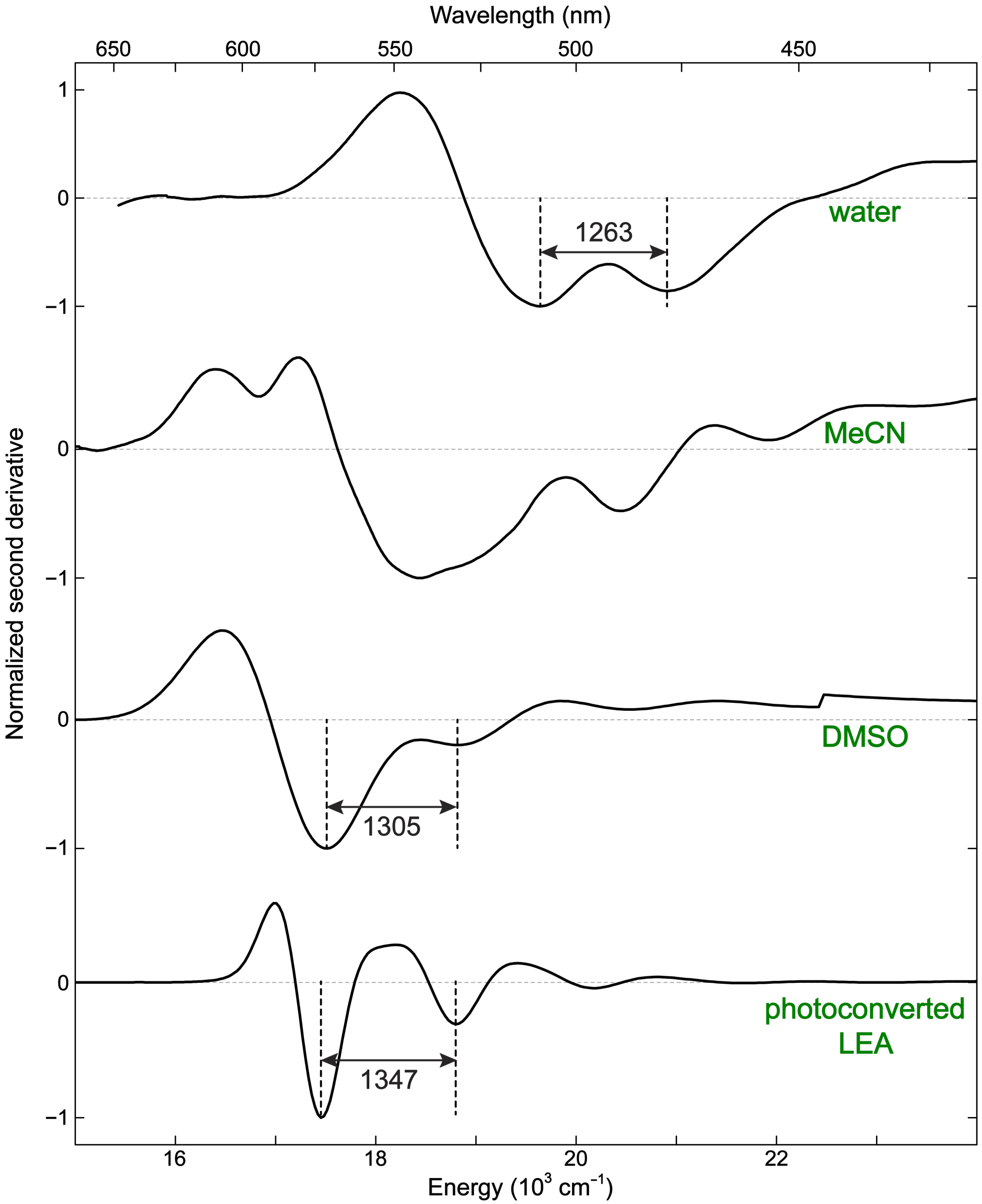

Figure S10. Second-derivative analysis of the electronic absorption spectra for the anionic Kaede chromophore in different solvents and the photoconverted LEA protein. The vibronically coupled mode frequency is estimated by the difference of the 0–0 and 0–1 transition peak energies (denoted by two vertical dashed lines) which are clearly resolvable for cases like the model chromophore in water or DMSO (Figure 10d), and the photoconverted LEA (red form, see Figure 10b in main text).

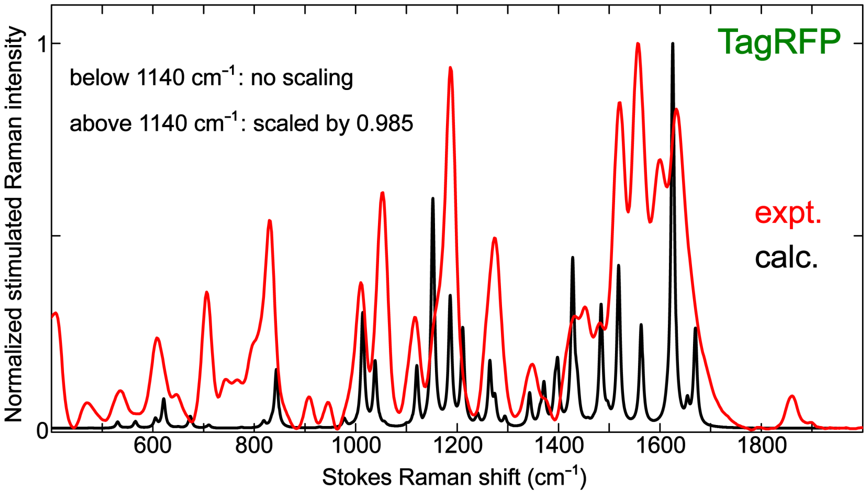

Figure S11. Comparison between the experimental and calculated Raman spectra of TagRFP. See Materials and Methods in main text for details of the experimental conditions and calculation methods. The frequency scaling factor is shown in the inset. The peak width of the calculated spectrum (black) is set at 8 cm^{-1} for a visual comparison with the experimentally observed spectrum (red).

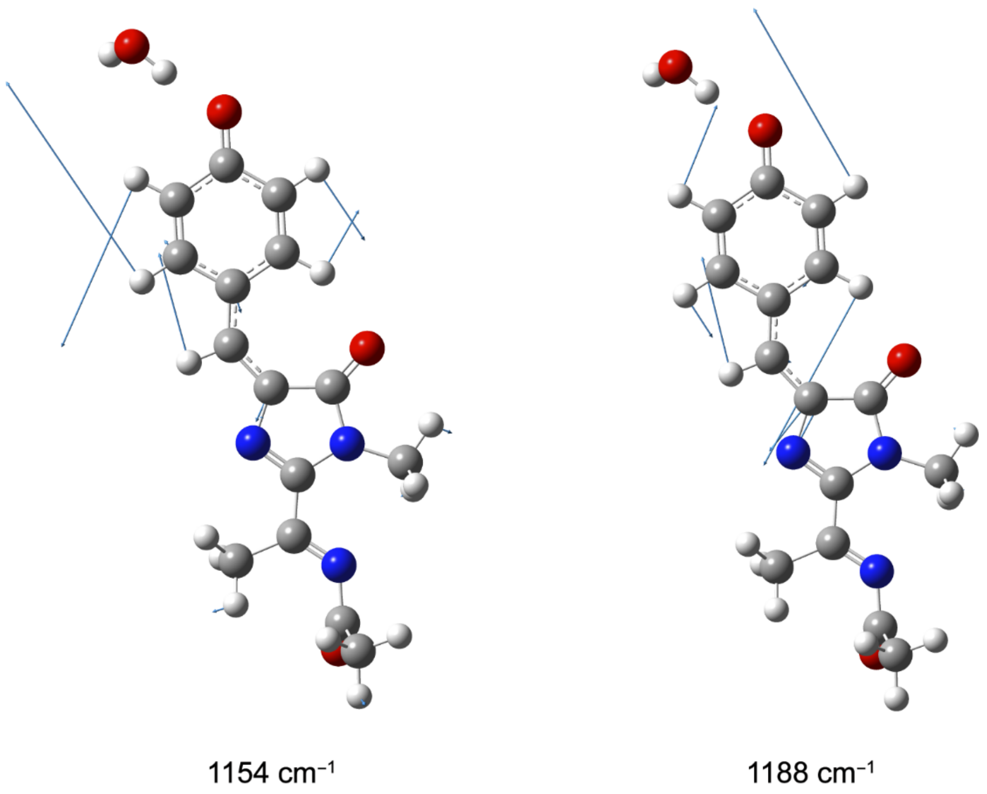

Figure S12. Motions of the H-rocking modes at ~1150–1200 cm^{-1} in TagRFP. See Materials and Methods in main text for details of the experimental conditions and calculation methods. The atomic/nuclear displacements mainly involving the P-ring and methine-bridge H's are depicted in blue arrows for the calculated vibrational normal modes of the deprotonated (anionic) chromophore with an adjacent water molecule at the phenolate end (see computational details in main text Section 3.4).

2. Supplementary Tables

Table S1. Mode assignment for the anionic *p*-HBI with –H and –Me substituents in water.[a]

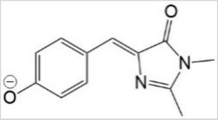		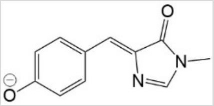		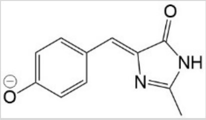		Vibrational motions[e]
Expt.	Calc.[b]	Expt.	Calc.[c]	Expt.	Calc.[d]	
–	–	–	–	516	509	**P**(τ), **I**(τ_{N-H})
521	522	–	–	–	–	**P**(β), **I**(δ_{C-H})
–	–	563	560	–	–	**I**(β, δ_{C-H}, ρ_{C-H}), **P**(β)
612	605	–	–	611	599	**I**(δ_{C-H}, β), **P**(β)
–	–	–	–	710	697	**I**(β), **P**(β)
–	–	744	745	–	–	**I**(β)
765	766	–	–	–	–	**I**(o), **P**(β)
852	847	838	840	–	–	**P**(o)
–	–	–	–	852	847	**P**(β)
932	935	–	–	–	–	**I**(δ_{C-H}, β)
–	–	–	–	992	996	**P**(τ)
1031	1037	1019	1017	–	–	**I**(δ_{C-H}, β), **B**(ρ_{C-H}), **P**(ρ_{C-H})
–	–	–	–	1109	1118	**P**(ρ_{C-H})
1164	1161	1161	1160	1159	1169	**P**(ρ_{C-H}), **B**(ρ_{C-H})
–	–	–	–	1202	1201	**P**(ρ_{C-H}), **B**(ρ_{C-H}), **I**(β, ρ_{N-H}, δ_{C-H})
		1230	1238	–	–	**B**(ρ_{C-H}), **I**(ρ_{C-H}, δ_{C-H}, ν_{C-N}, β), **P**(ρ_{C-H})
1238	1247	–	–	–	–	**P**(ν_{C-C}, ρ_{C-H}), **B**(ρ_{C-H}), **I**(δ_{C-H})
–	–	–	–	1263	1258	**I**(δ_{C-H}, ρ_{N-H}), **P**(ρ_{C-H})
1302	1292	1272	1272	1296	1290	**I**(ν_{C-N}, β, δ_{C-H}), **P**(ρ_{C-H}), **B**(ρ_{C-H})
–	–	1317	1319	–	–	**P**(ν_{C-C}, ρ_{C-H}), **B**(ρ_{C-H})
1363	1362	1359	1363	1358	1360	**P**($\nu_{C=O}$, ρ_{C-H}), **B**(ρ_{C-H}), **I**(ν_{C-N}, δ_{C-H})
1434	1449	1427	1436	1423	1450	**I**(δ_{C-H}, ν_{C-N})
1496	1492	1492	1491	1490	1492	**P**($\nu_{C=C}$, ν_{C-C}, ρ_{C-H}), **B**($\nu_{C=C}$)
**1548**	1546	**1541**	1542	**1554**	1549	**P**($\nu_{C=C}$, $\nu_{C=O}$, ρ_{C-H}), **B**($\nu_{C=C}$, ν_{C-C}, ρ_{C-H}), **I**($\nu_{C=N}$, $\nu_{C=O}$, δ_{C-H})[f]
1578	1585	1566	1560	1583	1589	**P**($\nu_{C=C}$, ρ_{C-H}), **I**($\nu_{C=N}$, $\nu_{C=O}$, δ_{C-H})
1623	1625	1615	1621	1627	1623	**P**($\nu_{C=C}$, $\nu_{C=O}$), **B**($\nu_{C=C}$), **I**($\nu_{C=O}$, $\nu_{C=N}$)

[a] The chromophores are deprotonated in basic aqueous solution with 0.01M NaOH. The frequency is in cm^{-1}. The experimental spectra are displayed in Figure 3 in main text (with Raman pump center wavelength of 509 nm).

[b] The calculated Raman mode frequencies are scaled by 0.985 and unscaled for the modes above and below 1075 cm^{-1}, respectively (see Figure S1a).

[c] The calculated Raman mode frequencies are scaled by 0.985 and unscaled for the modes above and below 1075 cm^{-1}, respectively (see Figure S1b).

[d] The calculated Raman mode frequencies are scaled by 0.985 and unscaled for the modes above and below 1325 cm^{-1}, respectively (see Figure S1c).

[e] Abbreviations for the chromophore's (1) structural moieties: **P** (phenolate ring), **I** (imidazolinone ring), and **B** (methine bridge); (2) vibrational normal mode motions: ν (stretching), δ (bending), ρ (in-plane rocking), β (ring deformation), o (ring breathing), and τ (out-of-plane twisting). We note that the bending motions of $–CH_3$ groups highlighted in gray (i.e., $\delta_{C–H}$) should become C–H/N–H rocking and bending of the unchanged $–CH_3$ group when the other $–CH_3$ is replaced with –H.

[f] See Figure 3 right panels in main text for illustration of the pertinent atomic displacements for this Raman marker band (with strongest intensity across the spectral detection window).

Table S2. Mode assignment for the anionic *p*-HBDI in two organic solvents.[a]

MeCN		DMSO		Vibrational motions[d]
Expt.	Calc.[b]	Expt.	Calc.[c]	
513	515	508	515	**P**(β), **I**(δ_{C-H})
603	604	602	604	**I**(δ_{C-H}, β), **P**(β)
713	712	712	712	**I**(δ_{C-H}, β), **P**(β)
758	754	757	754	**I**(o), **P**(β)
833	827	830	827	**P**(o)
917	919	921	919	**I**(δ_{C-H}, β), **B**(ρ_{C-H})
1023	1022	1022	1022	**I**(δ_{C-H}, β), **B**(ρ_{C-H}), **P**(ρ_{C-H})
1163	1159	1175	1159	**P**(ρ_{C-H})
1238	1246	1239	1246	**B**(ρ_{C-H}) , **P**(ν_{C-C}, ρ_{C-H}), **I**(δ_{C-H}, β)
1294	1293	1295	1293	**I**(ν_{C-N}, β, δ_{C-H}), **P**(ρ_{C-H}), **B**(ρ_{C-H})
1355	1363	1353	1363	**I**(ν_{C-N}, δ_{C-H}), **P**(ρ_{C-H}), **B**(ρ_{C-H})
1444	1450	1451	1449	**I**(δ_{C-H}, ν_{C-N})
1494	1501	1487	1501	**P**($\nu_{C=C}$, ν_{C-C}, ρ_{C-H}), **B**($\nu_{C=C}$, ρ_{C-H})
**1547**	1545	**1542**	1545	**P**($\nu_{C=C}$, $\nu_{C=O}$, ρ_{C-H}), **B**($\nu_{C=C}$, ν_{C-C}, ρ_{C-H}), **I**($\nu_{C=O}$, $\nu_{C=N}$, δ_{C-H})
1583	1583	1576	1583	**I**($\nu_{C=N}$, $\nu_{C=O}$, δ_{C-H}), **P**($\nu_{C=C}$, ρ_{C-H})
1624	1623	1618	1623	**P**($\nu_{C=C}$, $\nu_{C=O}$), **B**($\nu_{C=C}$), **I**($\nu_{C=O}$, $\nu_{C=N}$)
1659	1653	1654	1653	**I**($\nu_{C=O}$), **B**($\nu_{C=C}$)

[a] The chromophore is deprotonated in MeCN (acetonitrile) and DMSO (dimethyl sulfoxide) with 0.05–0.1% DBU (1,8-diazabicyclo[5.4.0]undec-7-ene) by volume. The frequency is in cm^{-1}. The experimental spectra are displayed in Figure 5b in main text (with Raman pump center wavelength of 540 nm).

[b] The calculated frequencies are scaled by 0.985 and unscaled for the modes above and below 700 cm^{-1}, respectively (see Figure S2a).

[c] The calculated frequencies are scaled by 0.985 and unscaled for the modes above and below 700 cm^{-1}, respectively (see Figure S2b).

[d] Abbreviations for the chromophore's (1) structural moieties: **P** (phenolate ring), **I** (imidazolinone ring), and **B** (methine bridge); (2) vibrational normal mode motions: ν (stretching), δ (bending), ρ (in-plane rocking), β (ring deformation), o (ring breathing), and τ (out-of-plane twisting).

Table S3. Mode assignment for the deprotonated chromophores of mPapaya1, mKO2, mOrange2, and mCherry. [a]

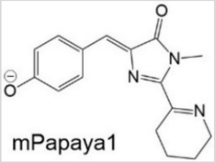		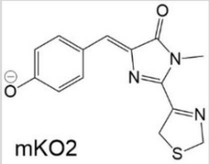		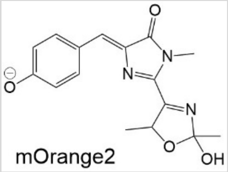		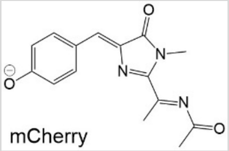		Vibrational motions[f]
Expt.	Calc.[b]	Expt.	Calc.[c]	Expt.	Calc.[d]	Expt.	Calc.[e]	
613	–	617	–	621	–	–	–	–[g]
655	–	663	–	652	–	–	–	–[g]
–	–	–	–	–	–	612	598	**I**(β, δ_{C-H}), **P**(β), **EX**($\tau_{C=N}$, δ_{C-H})
–	–	–	–	–	–	648	624	**EX**($\tau_{C=N}$, δ_{C-H}), **P**(β)
844	848	840	836	841	847	835	846	**P**(o), **I**(β, δ_{C-H})
1059	1072	1052	1039	1047	1040	1054	1041	**I**(δ_{C-H}), **EX**(δ_{C-H})
1178	1180	1173	1176	1173	1178	1173	1181	**P**(ρ_{C-H}), **B**(ρ_{C-H}), **I**(ν_{C-N})
1270	1268	1261	1262	1264	1273	1255	1250	**P**(ρ_{C-H}, β), **B**(ρ_{C-H}), **I**(δ_{C-H}, β), **EX**(δ_{C-H})
1334	1351	–	–	1330	1342	1331	1320	**EX**(δ_{C-H}), **P**(ρ_{C-H}), **B**(ρ_{C-H})
–	–	1328	1318	–	–	–	–	**I**(ν_{C-N}, δ_{C-H}, β), **EX**(δ_{C-H}), **P**(ρ_{C-H}), **B**(ρ_{C-H})
1367	1359	1365	1360	1357	1363	1363	1350	**P**(ρ_{C-H}, $\nu_{C=O}$), **B**(ρ_{C-H}), **EX**(δ_{C-H}), **I**(ν_{C-N})
1400	1383	1400	1397	1398	1400	1401	1394	**P**($\nu_{C=O}$, ρ_{C-H}), **B**(ρ_{C-H}), **I**(ν_{C-N}, δ_{C-H})
1490	1480	1488	1488	1485	1488	1473	1480	**P**($\nu_{C=C}$, ν_{C-C}, ρ_{C-H}), **B**($\nu_{C=C}$, ρ_{C-H}), **I**($\nu_{C=N}$, δ_{C-H})
1519	1509	1522	1517	1517	1517	1510	1510	**P**($\nu_{C=O}$, ρ_{C-H}, β), **I**($\nu_{C=N}$, δ_{C-H}, β)
**1558**	1555	**1558**	1565	**1556**	1563	**1561**	1559	**P**($\nu_{C=C}$, $\nu_{C=O}$, ρ_{C-H}), **B**($\nu_{C=C}$, ν_{C-C}, ρ_{C-H}), **I**($\nu_{C=O}$, $\nu_{C=N}$, δ_{C-H})
–	–	1606	1619	1604	1616	1603	1611	**P**($\nu_{C=C}$, $\nu_{C=O}$, ρ_{C-H}), **B**($\nu_{C=C}$, ρ_{C-H})
1622	1635	1633	1642	–		–	–	**EX**($\nu_{C=N}$), **I**($\nu_{C=O}$)

–	–	–	–	1635	1628	–	–	**EX**($\nu_{C=N}$), **I**($\nu_{C=O}$), **B**($\nu_{C=C}$), **P**($\nu_{C=C}$, $\nu_{C=O}$)
–	–	–	–	–	–	1637	1630	**EX**($\nu_{C=N}$, $\nu_{C=O}$), **I**($\nu_{C=O}$)

[a] The frequency is in cm^{-1}. The experimental spectra are displayed in Figure 8b in main text with Raman pump center wavelengths of 564, 616, 600, and 638 nm for mPapaya1, mKO2, mOrange2, and mCherry, respectively.

[b] The calculated frequencies are scaled by 0.98 and unscaled for the modes above and below 1340 cm^{-1}, respectively (see Figure S6a).

[c] The calculated frequencies are scaled by 0.985 and unscaled for the modes above and below 1350 cm^{-1}, respectively (see Figure S6b).

[d] The calculated frequencies are scaled by 0.985 and unscaled for the modes above and below 1340 cm^{-1}, respectively (see Figure S6c).

[e] The calculated frequencies are scaled by 0.985 and unscaled for the modes above and below 1140 cm^{-1}, respectively (see Figure S6e).

[f] Abbreviations for the chromophore's (1) structural moieties: **P** (phenolate ring), **I** (imidazolinone ring), **B** (methine bridge), and **EX** (extended ring or acylimine moiety); (2) vibrational normal mode motions: ν (stretching), δ (bending), ρ (in-plane rocking), β (ring deformation), o (ring breathing), and τ (out-of-plane twisting).

[g] There are no matching modes with close frequencies or prominent intensity. Higher-level calculations with more advanced theory and expanded basis sets may be needed to better accommodate the environmental effect in modulating the chromophore vibrational motions.

Table S4. Mode assignment for the deprotonated *trans* chromophore of KFP1. [a]

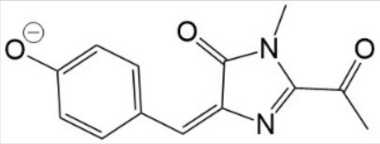		Vibrational motions [d]
Expt.	Calc. [b]	
752	–	– [c]
812	819	**P**(β), **I**(β, δ_{C-H})
900	915	**I**(β, δ_{C-H}), **P**(β), **B**(ρ_{C-H})
1008	–	– [c]
1120	1120	**P**(ρ_{C-H}), **B**(ρ_{C-H})
1150	1151	**P**(ρ_{C-H}), **B**(ρ_{C-H}), **I**(ν_{C-N})
1189	1188	**P**(ρ_{C-H}), **B**(ρ_{C-H}), **I**(ν_{C-N})
1272	1275	**P**(ρ_{C-H}, β), **B**(ρ_{C-H}), **I**(δ_{C-H})
1349	1357	**A**(δ_{C-H})
1373	1368	**A**(δ_{C-H}), **I**(δ_{C-H}, β), **P**(ρ_{C-H}), **B**(ρ_{C-H})
1410	1422	**I**(δ_{C-H}, $\nu_{C=N}$, β), **B**($\nu_{C=C}$, ρ_{C-H}), **P**(ρ_{C-H})
1470	1481	**P**($\nu_{C=C}$, ν_{C-C}, ρ_{C-H}), **B**($\nu_{C=C}$, ρ_{C-H}), **I**($\nu_{C=N}$, δ_{C-H})
1512	1517	**P**($\nu_{C=O}$, ρ_{C-H}, β), **I**($\nu_{C=N}$, δ_{C-H}, β), **B**($\nu_{C=C}$, ρ_{C-H})
**1563**	1562	**P**($\nu_{C=C}$, $\nu_{C=O}$, ρ_{C-H}), **B**($\nu_{C=C}$, ν_{C-C}, ρ_{C-H}), **I**($\nu_{C=O}$, $\nu_{C=N}$, δ_{C-H})
1605	1614	**P**($\nu_{C=C}$, $\nu_{C=O}$, ρ_{C-H}), **B**($\nu_{C=C}$, ρ_{C-H})
1636	1631	**A**($\nu_{C=O}$, δ_{C-H}), **I**($\nu_{C=O}$), **B**($\nu_{C=C}$, ν_{C-C}, ρ_{C-H}), **P**(ρ_{C-H})
1658	1663	**A**($\nu_{C=O}$, δ_{C-H}), **I**($\nu_{C=O}$), **B**($\nu_{C=C}$, ν_{C-C}, ρ_{C-H}), **P**($\nu_{C=C}$, ρ_{C-H})

[a] The frequency is in cm^{-1}. The experimental spectrum is displayed in Figure 8b (main text) with Raman pump center wavelength of 640 nm for KFP1.

[b] The calculated frequencies are scaled by 0.985 and unscaled for the modes above and below 1140 cm^{-1}, respectively (see Figure S6d).

[c] There are no matching modes with close frequencies or prominent intensity.

[d] Abbreviations for the chromophore's (1) structural moieties: **P** (phenolate ring), **I** (imidazolinone ring), **B** (methine bridge), and **A** (acetyl); (2) vibrational motions: ν (stretching), δ (bending), ρ (in-plane rocking), β (ring deformation), o (ring breathing), and τ (out-of-plane twisting).

Table S5. Mode assignment for the deprotonated Kaede model chromophore and the photoconverted LEA chromophore.[a]

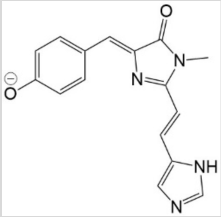						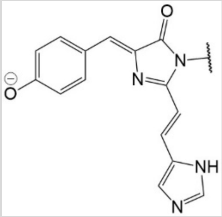	Vibrational motions[e]
Expt. (H_2O)	Calc.[b]	Expt. (MeCN)	Calc.[c]	Expt. (DMSO)	Calc.[d]	Expt.	
592	591	588	591	588	591	619	**I**(β), **P**(β)
–	–	–	–	–	630	641	**P**(β)
857	853	847	849	843	849	851	**P**(o), **I**(β, δ_{C-H})
968	980	958	977	951	978	937	**I**(δ_{C-H}, β)
1036	1038	1029	1012	1032	1012	1026	**I**(δ_{C-H}, β), **B**(ρ_{C-H}), **P**(ρ_{C-H})
–	1098	1093	1099	1085	1099	1083	**SI**(ρ_{N-H}, ρ_{C-H})
1167	1159	1161	1147	1158	1147	1174	**P**(ρ_{C-H}), **B**(ρ_{C-H})
1238	1241	1236	1235	1239	1235	1259	**P**(ρ_{C-H}, β), **B**(ρ_{C-H}), **I**(δ_{C-H}, β), **SI**(ρ_{C-H}, ρ_{N-H})
1286	1287	1280	1281	1277	1281	1290	**SI**(ρ_{C-H})
1310	1322	1307	1316	1310	1316	1340	**SI**(ρ_{C-H}), **I**(δ_{C-H}, ν_{C-N}, β), **P**(ρ_{C-H})
1365	1357	1354	1353	1354	1353	1368	**P**(ρ_{C-H}, $\nu_{C=O}$), **B**(ρ_{C-H}), **I**(ν_{C-N})
1459	1479	1452	1476	1452	1476	1464	**P**($\nu_{C=C}$, ν_{C-C}, ρ_{C-H}), **B**(ρ_{C-H}), **I**(δ_{C-H}, $\nu_{C=N}$)
**1495**	1507	**1487**	1504	**1483**	1504	1493	**P**($\nu_{C=O}$, ρ_{C-H}), **B**(ν_{C-C}, ρ_{C-H}), **I**($\nu_{C=N}$, δ_{C-H}), **SI**(ρ_{C-H})
–	1545	1521	1537	1521	1537	1522	**SI**($\nu_{C=C}$, ν_{C-C}, $\nu_{C=N}$, ρ_{C-H}, ρ_{N-H}), **P**($\nu_{C=C}$, $\nu_{C=O}$, ρ_{C-H}), **B**($\nu_{C=C}$, ν_{C-C}, ρ_{C-H}), **I**($\nu_{C=N}$, $\nu_{C=O}$)
1567	1562	1565	1553	1559	1553	1558	**SI**($\nu_{C=C}$, ν_{C-C}, ρ_{C-H}, ρ_{N-H}), **P**($\nu_{C=C}$, $\nu_{C=O}$, ρ_{C-H}), **B**($\nu_{C=C}$, ν_{C-C}, ρ_{C-H}), **I**($\nu_{C=O}$)
1631	1634	1628	1626	1625	1626	1628	**SI**($\nu_{C=C}$, ν_{C-C}, ρ_{C-H}, ρ_{N-H})

[a] The model chromophore is deprotonated in basic aqueous solution with 1 mM NaOH and in organic solvents (MeCN and DMSO) with 0.05–0.1% DBU (1,8-diazabicyclo[5.4.0]undec-7-ene) by volume ratio (v/v). The frequency is in cm^{-1}. The experimental spectra for the deprotonated Kaede model chromophore are displayed in Figure 10e (main text) with Raman pump center wavelengths of 600 and 640 nm in water and MeCN or DMSO, respectively. The experimental spectrum for the photoconverted LEA chromophore (red form) is displayed in Figure 10c (main text) with Raman pump center wavelength of 610 nm.

[b] The calculated frequencies are scaled by 0.98 and unscaled for the modes above and below 1075 cm^{-1}, respectively (see Figure S9a).

[c] The calculated frequencies are scaled by 0.975 and unscaled for the modes above and below 900 cm^{-1}, respectively (see Figure S9b).

[d] The calculated frequencies are scaled by 0.975 and unscaled for the modes above and below 900 cm^{-1}, respectively (see Figure S9c).

[e] Abbreviations for the chromophore's (1) structural moieties: **P** (phenolate ring), **I** (imidazolinone ring), **B** (methine bridge), and **SI** (styryl imidazole); (2) vibrational normal mode motions: ν (stretching), δ (bending), ρ (in-plane rocking), β (ring deformation), o (ring breathing), and τ (out-of-plane twisting). The most prominent experimental peaks between ca. 1480–1495 cm^{-1} for the Kaede model chromophore in various solvents (see Figure 10e) are bolded in this table.

Table S6. Mode assignment for the deprotonated *trans* chromophore of TagRFP. [a]

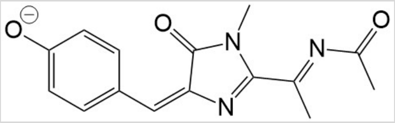		Vibrational motions [c]
Expt. (H_2O)	Calc. [b]	
607	605	**P**(β), **EX**(δ_{C-H})
649	621	**EX**($\tau_{C=N}$, δ_{C-H})
706	711	**I**($\tau_{C=N}$, τ_{C-N}), **EX**(δ_{C-H})
832	844	**P**(o), **EX**(δ_{C-H})
1011	1014	**EX**(δ_{C-H})
1053	1038	**I**(δ_{C-H}, β), **EX**(δ_{C-H}), **B**(ρ_{C-H}), **P**(ρ_{C-H})
1118	1120	**P**(ρ_{C-H}, β), **B**(ρ_{C-H})
1154 [d]	1152	**P**(ρ_{C-H}), **B**(ρ_{C-H}), **I**(ν_{C-N})
1188 [d]	1186	**P**(ρ_{C-H}), **B**(ρ_{C-H}), **I**(ν_{C-N})
1275	1265	**P**(ρ_{C-H}, β), **B**(ρ_{C-H}), **I**(ν_{C-N}, δ_{C-H})
1349	1343	**P**($\nu_{C=C}$, ν_{C-C}, ρ_{C-H}), **B**(ρ_{C-H})
1520	1518	**P**($\nu_{C=O}$, ρ_{C-H}, β), **I**($\nu_{C=N}$, δ_{C-H}, β), **B**(ρ_{C-H})
**1556**	1563	**P**($\nu_{C=C}$, $\nu_{C=O}$, ρ_{C-H}), **B**($\nu_{C=C}$, ν_{C-C}, ρ_{C-H}), **I**($\nu_{C=O}$, $\nu_{C=N}$, δ_{C-H})
1599	1614	**P**($\nu_{C=C}$, $\nu_{C=O}$, ρ_{C-H}), **B**($\nu_{C=C}$, ρ_{C-H})
1633	1626	**EX**($\nu_{C=N}$, $\nu_{C=O}$, δ_{C-H}), **I**($\nu_{C=O}$, $\nu_{C=N}$), **B**($\nu_{C=C}$, ν_{C-C}, ρ_{C-H}), **P**($\nu_{C=C}$, ρ_{C-H})

[a] The frequency is in cm^{-1}. The experimental spectrum for the deprotonated chromophore of TagRFP is displayed in Figure 12c (main text) with Raman pump center wavelength of 616 nm.

[b] The calculated frequencies are scaled by 0.985 and unscaled for the modes above and below 1140 cm^{-1}, respectively (see Figure S11).

[c] Abbreviations for the chromophore's (1) structural moieties: **P** (phenolate ring), **I** (imidazolinone ring), **B** (methine bridge), and **A** (acetyl); (2) vibrational motions: ν (stretching), δ (bending), ρ (in-plane rocking), β (ring deformation), o (ring breathing), and τ (out-of-plane twisting).

[d] The motions for these two adjacent vibrational modes differ in the phase of the rocking motions between the P-ring H and bridge H atoms (see Figure S12 above for the normal mode illustration).
